# Differential stability of bacterial photosynthetic apparatus of *Rhodobacter alkalitolerans* strain JA916^T^ under alkaline and light environment

**DOI:** 10.3389/fmicb.2024.1360650

**Published:** 2024-03-14

**Authors:** Mohammad Yusuf Zamal, Saikiran Madireddi, Nageswara Rao Mekala, Venkata Ramana Chintalapati, Rajagopal Subramanyam

**Affiliations:** Department of Plant Science, School of Life Sciences, University of Hyderabad, Gachibowli, Telangana, India

**Keywords:** light-harvesting complexes (LH1 andLH2), high light, intracytoplasmic membranes (ICMs), reaction center-light-harvesting complex (RC-LH1), *Rhodobacter alkalitolerans* strain JA916T

## Abstract

In purple bacteria, photosynthesis is performed by densely packed pigment-protein complexes, including the light-harvesting complexes known as RC-LH1 and LH2, with carotenoids to assist in the functioning of photosynthesis. Most photosynthetic bacteria are exposed to various abiotic stresses such as light, temperature, alkalinity–acidity, and salinity. *Rhodobacter (R.) alkalitolerans* was discovered from the alkaline pond; here, we report the comparative study of the photosynthetic apparatus of *R. alkalitolerans* in various light intensities in relation to its high pH tolerance ability. With increased light intensity, the stability of photosystem complexes decreased in normal pH (npH pH 6.80 ± 0.05) conditions, whereas in high pH (hpH pH 8.60 ± 0.05), acclimation was observed to high light. The content of bacteriochlorophyll *a*, absorbance spectra, and circular dichroism data shows that the integrity of photosystem complexes is less affected in hpH compared with npH conditions. Large pore blue native polyacrylamide gel electrophoresis of photosystem protein complexes and sucrose density gradient of n-dodecyl β-D-maltoside solubilized intracytoplasmic membranes show that LH2 is more affected in npH than in hpH, whereas RC-LH1 monomer or dimer has shown interplay between monomer and dimer in hpH, although the dimer and monomer both increased in npH. Increased content and expression level of ATPase protein complex and subunit—“c” of ATPase, fast relaxation kinetics of p515, and relatively higher membrane lipid content in hpH along with less photooxidative stress and subsequently lesser superoxide dismutase activity exemplify photoprotection in hpH. Furthermore, the increased expression levels of antiporter NhaD in hpH signify its role in the maintenance of homeostatic balance in hpH.

## Introduction

Photosynthesis is the primary source of energy nearly for all sorts of life forms on Earth. It starts with the absorption of sunlight and subsequently converts the light energy to different forms of hydrocarbon energy. In purple bacteria, photosynthesis is performed by a properly oriented network of densely packed pigment-protein complexes known as light-harvesting comlex2 (LH2) and light harvesting complex1 (LH1), capturing photons and transferring the energy to the reaction center (RC) (van Grondelle et al., 1994; Niedzwiedzki et al., 2018). Electron passes from RC to the cytochrome (Cyt) bc1 via quinone/quinol exchange at the Qb site of the RC (Okamura et al., 2000; Francia et al., 2004). Light harvesting complexes comprise roughly circularly (elliptically) arranged α-helixes with bound carotenoids and bacterial chlorophyll (BChl) pigments (Bahatyrova et al., 2004). The α-helixes comprise α and β proteins forming the oligomeric light-harvesting complexes LH2 and LH1, and LH1 also encompasses the reaction center (RC), making the complex RC-LH1 (Roszak et al., 2003; Semchonok et al., 2012). RC-LH1 is found in two forms: monomeric form, where the RC is surrounded by C-shaped LH1, and dimeric form, where the S-shaped LH1 complex aggregation surrounds the two reaction centers (Jungas et al., 1999; Crouch and Jones, 2012). The LH2 and RC-LH1 complexes follow a certain pathway of electron transfer in order to generate reducing equivalents, wherein the photon absorbed by the LH2 is transferred to the LH1 and from LH1 to the reaction center, where charge separation takes place between the donor and acceptor molecules. The electron transfer pathway is constituted by a dimer of bacterial chlorophyll, bacterial pheophytin (BPhe), and Cyt bc1 complex, making a cyclic electron flow (Meyer and Donohue, 1995; Jungas et al., 1999). The purple non-sulfur gram-negative bacteria house LH1-RC and LH2 by invaginating the bacterial cytoplasmic membranes, leading to the formation of vesicular membranous structures known as intracytoplasmic membranes (ICMs), also called chromatophores (Niederman, 2013). Some of the ICMs are attached to the plasma membrane as they are developing, whereas matured ICMs are now in the cytoplasm as the development of the cell proceeds (Naylor et al., 1999; Adams and Hunter, 2012; Niederman, 2013).

*Rhodobacter* is a genus of metabolically versatile bacteria that can grow in aerobic and anaerobic conditions. *Rhodobacter* has been used to study photosynthesis, hydrogen production, and bioplastic polyhydroxy butyrate (PHB) production (Mougiakos et al., 2019). In the present study, the model organism, *Rhodobacter (R.) alkalitolerans* strain JA916^T^, can grow in alkaline culture conditions and discovered in an alkaline pond (Gandham et al., 2018). The strategies adopted by bacteria, while grown in alkaline conditions, include (a) increased metabolic acid production through amino acid deaminases and sugar fermentation; (ii) increased ATP synthase that couples H^+^ entry to ATP generation; (iii) changes in cell surface properties, and (iv) increased expression and activity of monovalent cation/proton antiporters (Padan et al., 2005). Since *R. alkalitolerans* is a photosynthetic bacterium that utilizes photosynthesis to produce ATP, it could pave the way to understanding the relation between alkaline (high pH) tolerance and production of ATP by photosynthetic machinery and its own functioning in relation to alkaline conditions. Many bacteria have been discovered to have adoptive strategies to grow in extreme environmental conditions, such as alkaliphilic acidophiles halophiles which use several antiporters, such as monocationic and dicationic proton antiporters, e.g., sodium/proton antiporters and calcium/proton antiporters (Padan et al., 2005; Preiss et al., 2015).

Previously, many species from the genus *Rhodobacter* have been studied for its basic mechanism of photosynthesis and its photosynthetic membrane protein complexes, i.e., RC-LH1 monomer, dimer, and LH2 housed in chromatophore/intracytoplasmic membranes (Ng et al., 2011; Crouch and Jones, 2012). They have also been studied at biophysical level to investigate the electron transfer efficiency using femtosecond transient absorption spectroscopy and metabolite study (Yen et al., 2010; Niedzwiedzki et al., 2018). Many species of purple non-sulfur photosynthetic bacteria such as *Rhodobacter sphaeroides 2.4.1*, *Rhodospirillum rubrum* (Hustede et al., 1993; Ryu et al., 2014), and *Rhodobacter capsulatus KU002* (Merugu et al., 2012) have been studied for the production of hydrogen gas and a bioplastic known as polyhydorxybutyrate (PHB). *R. alkalitolerans was* discovered from an alkaline pond, and being a photosynthetic organism and ability to grow in hpH, it promises the existence of interplay between homeostasis and photosynthesis, which can be investigated.

In nature, light intensity is never constant; as a result, photosynthetic organisms face extreme and low light intensity. Moreover, it is important to study how a bacterium that uses light to produce ATP while growing in alkaline conditions maintains the integrity of its photosynthetic machinery. Since it is a new photosynthetic bacterium, it has not been characterized by how it can cope with various stresses, especially high light and alkalinity. Thus, in the current study, we are trying to understand the effect of high light in relation to the high pH tolerance ability of *R. alkalitolerans* on the organization of photosynthetic complexes.

## Materials and methods

### Growth curve, calculation of generation time and pH measurement

For calculating the generation time, 8 mL screw-capped tubes were inoculated with 5% of inoculum (v/v) from mother cultures in the log phase. Triplicates of each bacterial culture were taken, and growth in terms of optical density was measured by taking reading at 660 nm. Generation time was calculated after plotting the growth curve (Willey et al., 2008; Tucker et al., 2010). pH of the culture was measured in all the conditions just before harvesting the cell using pH tutor of Eutech instruments.

### Culturing and harvesting of cells

Cultures were grown in Biebl and Pfennig’s medium in two pH conditions of pH 6.80 ± 0.05 and pH 8.60 ± 0.05 with sodium pyruvate (3 g/L) as carbon source and ammonium chloride as nitrogen source (0.4 g/L) in light/anaerobically at room temperature (25°C) in filled glass bottles with glass cork stoppers to avoid infiltration of air without any void space left as described previously (Lakshmi et al., 2011; Crouch and Jones, 2012; Gandham et al., 2018). The cultures were grown in three light intensities of 28–30 μmol photons m^−2^ s^−1^(optimum light), 250–255 μmol photons m^−2^ s^−1^, and 500 ± 5 μmol photons m^−2^ s^−1^ by inoculating 1.125 mL of inoculum culture in 300 mL of Biebl and Pfennig’s medium prepared in 25 mM Tris buffer medium, and the pH was set to 6.80 ± 0.05 with hydrochloric acid, and in hpH, the pH was reached to 8.60 ± 0.05 after adding all media components. Cells were harvested in the late log phase (~at OD of 1.4–1.5) (Scheuring et al., 2014) to get maximum number of intracytoplasmic membranes by centrifuging the cells at 15,000 × g for 20 min, washed with 20 mM HEPES pH 7.5, and then stored at −80°C until required.

### Harvesting of intracytoplasmic membranes

The pellet was washed with 20 mM of HEPES buffer with pH of 7.5. To harvest the ICMs cell in 15 ml HEPES buffer were sonicated on ice for 6 min at 30% amplitude by giving 45 s of relaxation and 15 min on (Tucker et al., 2010). Before sonication, protease inhibitor cocktail (sigma) was also added to avoid protein degradation. The cell lysate was ultracentrifuged in a 15/40% discontinuous sucrose density gradient at 50,000 × g in a Beckman Type SW32 Ti rotor at 4°C for 12 h. A pigmented band of ICM formed at the 15/40% interface and was collected using a fixed needle (Mothersole et al., 2016) and stored at −20°C and − 80°C for long storage. The membranes were solubilized with 1% n-dodecyl-beta-D-maltoside (β-DM) for 30 min, and the non-solubilized material was removed by centrifugation at 10,000 × g for 30 min (D'Haene et al., 2014). The supernatant was loaded in either BN-PAGE or in sucrose density gradient.

### Bacteriochlorophyll *a* estimation and carotenoid/BChl *a*

The content of BChl *a* and carotenoid was measured by resuspending the cell pellet in seven parts of acetone and two parts of methanol (v/v), and the absorbance readings were measured at 775 nm and 456 nm, respectively. The contents of BChl *a* and carotenoid were calculated as described (Cohen-Bazire et al., 1957). The carotenoid to Bach *a* ratio was also calculated (Supplementary Excel Sheet S1).

### Absorbance spectroscopy

Absorbance spectra of isolated intracytoplasmic membranes were measured in a quartz cuvette of 1 cm path length between 400 and 900 nm at room temperature (RT) at a protein concentration of 50 μg/mL using perkinelmer 1,500 UV–Visible spectrophotometer (Georgakopoulou et al., 2006). Protein concentrations were measured by the Bradford method (Quick Start Bradford 1x Dye Reagent, cat:5000205) using bovine serum albumin (BSA) as standard. The same method was used for all other protein estimations.

### Sucrose density gradient sedimentation

Sucrose density gradients were formed in transparent ultracentrifuge tubes by carefully layering five steps of 20, 21.3, 22.5, 23.8, and 25% (w/w) sucrose in 20 mM HEPES (pH 7.5) and 0.03% β-DM. Solubilized membrane proteins (300 μL at 1 μg/μL concentration) were loaded onto each gradient, and these were centrifuged in a Beckman coulter swing-out bucket rotor SW41Ti at 180,000 × g for 18 h at 4°C (D'Haene et al., 2014). For each growth condition, multiple gradients were run.

### Circular dichroism spectroscopy

Circular dichroism (CD) spectra of ICMs (in 20 mM HEPES buffer with 10 mM MgCl_2_) at protein concentration of 50 μg/mL were measured at room temperature using a Jasco J-1500 CD spectrometer and a 10-mm path length quartz cuvette with a protein concentration of 50 μg/mL. The NIR photomultiplier parameters were set: data pitch: 1 nm, bandwidth: 4 nm, response: 1 s. Two scans for each sample were collected at a speed of 100 nm/min (Southall et al., 2018), and each sample was in triplicate from three different biological replicates.

### Large pore blue native PAGE

The Blue native gel separation of photosynthetic complexes was carried out in gradient gel of 3.5–12% by solubilization of intracytoplasmic membranes (ICMs) in 1% β-DM along with the protease inhibitors 1 mM Amino Caproic Acid (ACA), 1 mM benzamidine hydrochloride, the gel with 50 mM ACA is run at 4°C with a constant current of 4 mA (Kügler et al., 1997; Madireddi et al., 2014).

### Identification of subunit protein of super-complexes separated by blue native-PAGE

Blue-native gel strips were carefully cut and stored at −20°C. BN strips were solubilized in laemmli buffer by gently shaking for 1 h with 6 M urea containing β-mercaptoethanol as a reducing agent. Solubilized BN strips were run with 15% SDS-urea gel. Individual protein subunits of the protein complex from BN-PAGE were spotted, excised, and given for identification by orbitrap high-resolution liquid chromatography-mass spectrometry (OHRLCMS, Agilent Technology USA), and subsequent quantitative studies were conducted in different treatments.

### Transmission electron microscopy

To investigate the morphology and number of ICMs in varying light intensities in relation to high pH tolerance, TEM imaging was performed. Harvested cells were washed with 50 mM potassium phosphate (KPi buffer) buffer pH 6.80, and primary fixation was performed with 2% glutaraldehyde Grade-I (Sigma) for 1 h at 4°C in the dark, and then, cells were washed three times with KPi buffer for 15 min each, post-fixed in KMNO_4_ (4% w/v) for 1 h, and washed with autoclaved distilled water five times each for 5 min. After this, cells were fixed with 2% (w/v) uranyl acetate for 1 h at room temperature (RT), and after washing with distilled water five times each for 5 min, cells were subjected to the dehydration step with ethanol (50, 70, 80, 90, and 95%) and four washes with 100% ethanol. The last two washes were performed with propylene oxide (≥ 99% from Sigma CAS: 75–56-9) two times each for 1 h. These cells were infiltrated with Spurr’s low-viscosity embedding medium, and blocks were prepared. Ultra-thin sections were cut and mounted on copper grids. Sections were stained with an alcoholic solution of 2% (w/v) uranyl acetate and Reynolds lead citrate stain (Fedotova and Zeilstra-Ryalls, 2014). The thin sections of cells were visualized using a Technai instrument.

### Electrochromic shift measurement

Fast relaxation kinetics measurements were performed in Dual PAM-100 (Walz) equipped with P515/P535 emitter detector module by saturating single turnover flash to intact cells of *R. alkalitolerans.* Cells at 1.4–1.5 OD were incubated in the dark for 1 h in the same culture medium before the measurement (Feniouk et al., 2002).

### RNA extraction, cDNA synthesis, and quantitative real-time PCR

Total RNA was extracted from STRN50-1KTspectrum ™ Plant Total RNA Kit, according to the manufacturer’s protocol. RNA concentration was calculated at 260/280 nm with a NanoDrop1000 spectrophotometer from Thermo Scientific. Single-stranded cDNA is synthesized from total RNA by cDNA synthesis kit (TAKARA) by priming at 65°C and reverse transcription for 1 h at 42°C in a 20 μL reaction mixture.

To investigate the expression level of some important genes, primers were designed for ATPase subunit “c” and antiporter NhaD based on the available genome sequence of closely related species *Rhodobacter sphaeroides ATH 2.4.1^T^ (X53853)* (Supplementary Table S1). Using *R. alkalitolerans,* cDNA genes were amplified, and the 2^**− Δ ΔCT**^ method was used to measure the accurate gene expression of target and housekeeping gene recA. RT-PCR was carried out in Eppendorf Mx3000P multiplex quantitative PCR system with SYBR Green PCR Master Mix (Kappa).

### Extraction and separation of polar lipids

To extract the polar lipids, 5 mg of lyophilized cells was taken. An extraction mixture of methanol-chloroform-water (1.1:0.9, v/v) was added to cells (De Leo et al., 2009), and phase separation was performed by centrifuging the mixture at 10,000 rpm for 6 min. The lower layer of chloroform was carefully collected, transferred to another fresh tube, and dried in a speed vacuum. Lipid dried was redissolved in 50 μL of chloroform and kept at −20°C until use. Total lipid extracts were analyzed by TLC on silica gel (20 × 20 cm, layer thickness 0.2 mm). The plate was developed with the solvent chloroform-methanol-acetic acid-water (85, 15, 10:3.5, v/v) and detected by iodine vapor. Lipid membrane bands were quantified by ImageJ software.

### Estimation of total ROS and analysis of SOD activity

To estimate the total amount of reactive oxygen species (ROS), cells from all the conditions were collected in logarithmic phase at equal OD of 1.4–1.5. Fluorescent dye 2′,7′-dichlorofluorescein diacetate (H_2_DCFDA) (Sigma–Aldrich) was used to quantify the total ROS. Cells were washed in culture medium and then incubated in the dark by adding 5 μM of dye for 1 h at room temperature. After incubation, the cells were washed in culture medium to remove excess dye. Fluorescence intensity was measured using a microplate reader (Tecan M250) at the excitation wavelength of 485 nm and emission at 530 nm (Devadasu et al., 2021).

In addition, to analyze the superoxide dismutase activity by native polyacrylamide gel electrophoresis, cells were lysed by sonication, and the supernatant was collected. Protein concentration was measured in all the samples by the Bradford method, and 20 μg of protein was loaded to check the type of superoxide dismutase expressed during the photoheterotrophic growth of bacteria. To visualize the SOD bands, gel was stained in 0.05 mM riboflavin, 0.1 mM nitro blue tetrazolium, and 1 mM EDTA, and 0.3% N,N,N′,N′-tetramethylethylenediamine made in 50 mM potassium phosphate buffer (pH 7.8) as described previously (Kho et al., 2006; Alves et al., 2020).

### Statistical analysis

All physiological and biochemical results are the average of at least three independent experiments performed separately. The results were analyzed by analysis of variance (ANOVA Tukey’s Test). Each data point averages three replicates, and error bars are represented as ±SE. Asterisks indicated the level of significance of hight light treated versus control (optimum light) with *P*-value style: GP: 0.1234 (ns), 0.0332(*), 0.0021(**), 0.0002 (***), and <0.0001 (****) respectively.

## Results

### Growth and generation time

**Figure 1:**
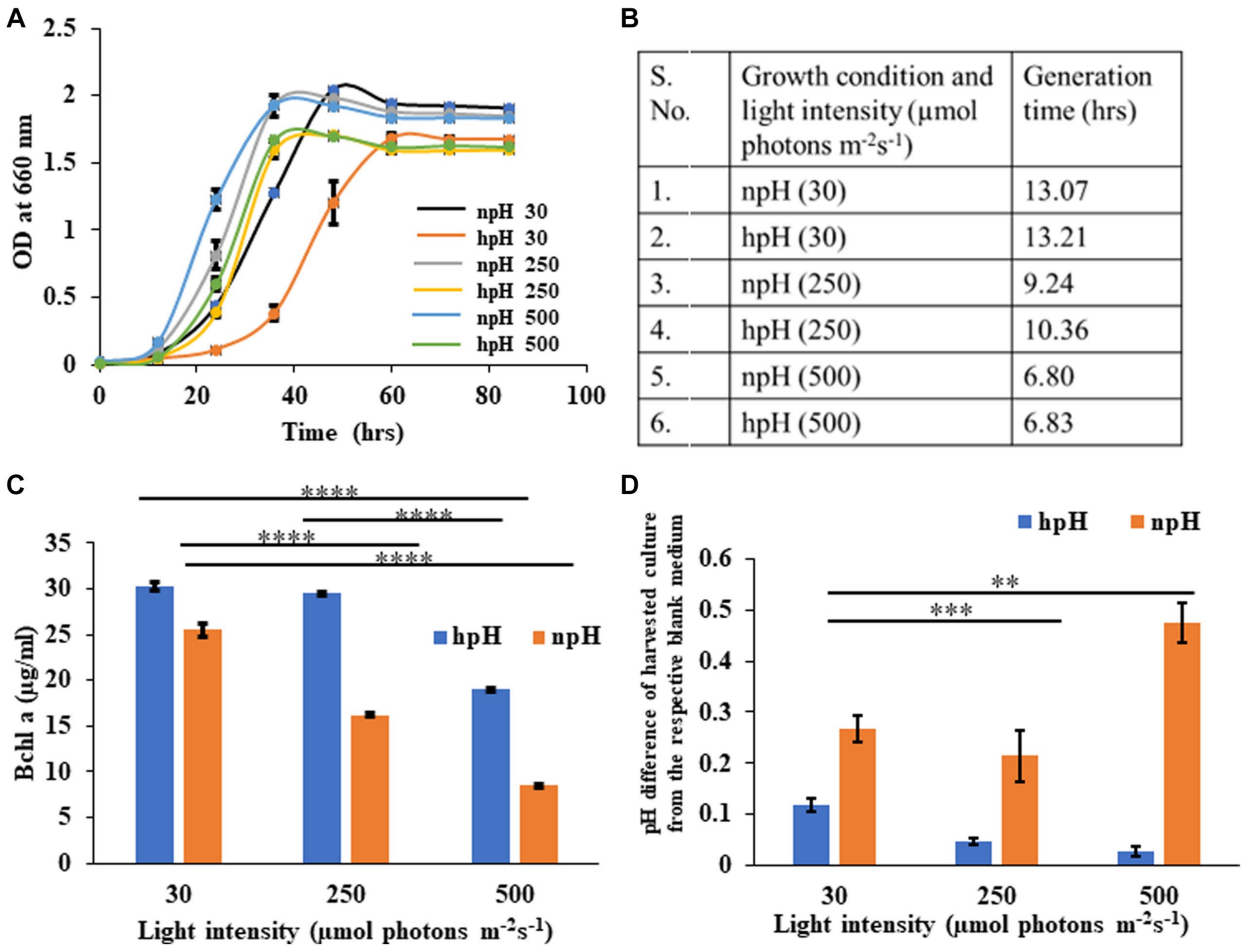
**(A)** Growth curve of *R. alkalitolerans* in alkaline (pH 8.60 ± 0.05, hpH) and at normal pH (pH 6.80±0.05, npH) in light intensities of 30 μmol photons m^−2^ s^−1^, 250 μmol photons m^−2^ s^−1^, and 500 μmol photons m^−2^ s^−1^. Cultures were grown anaerobically in 8 mL screw-capped tubes at room temperature (25°C). **(B)** Generation time: Calculation of generation time/doubling time at 30 μmol photons m^−2^ s^−1^, 250 μmol photons m^−2^ s^−1^, and 500 μmol photons m^−2^ s^−1^in npH and hpH culture media. **(C)** Comparative representation of the content of BChl *a* in hpH and npH conditions in all the three light conditions (30 μmol photons m^−2^ s^−1^, 250 μmol photons m^−2^ s^−1^, and 500 μmol photons m^−2^ s^−1^). **(D)** Measurement of pH difference of the culture from that of blank medium in hpH and npH culture grown at light intensity of 30, 250, and 500 μmol photons m^−2^ s^−1^. Asterisks indicated the level of significance of hight light (treated) versus control (normal light) with *p*-value style: GP: 0.1234 (ns), 0.0332(*), 0.0021(**), 0.0002 (***), and <0.0001 (****) respectively.

The growth curve of *R. alkalitolerans* in different light intensities in high pH and normal pH conditions was plotted (Figure 1A). There has been an effect of high pH on the attainment of the stationary phase. hpH grown cells entered the stationary phase around the optical density of 1.6, which took 40 h in high light (250 and 500 μmol photons m^−2^s^−1^), whereas at 30 μmol photons m^−2^s^−1^, it took nearly 60 h to enter the stationary phase. The npH grown cells took 1.9 optical density, which took 40 h to enter the stationary phase. The light affects the generation time as it decreases as light intensity increases (Figure 1B). Generation time remains almost the same at 30 μmol photons m^−2^s^−1^ being 13.07 hrs in npH and 13.21 hrs in hpH. At 250 μmol photons m^−2^s^−1^, 9.24 hrs in npH and 10.36 h in hpH, and at 500 μmol photons m^−2^s^−1^, 6.80 hrs in npH and 6.83 hrs in hpH.

### Bacteriochlorophyll *a* estimation and carotenoid to BChl *a* ratio

Bacteriochlorophyll *a* is one of the major pigments and, along with carotenoids, plays an essential role in bacterial photosynthesis, contributing to the assembly and structural stability of photosystem complexes. It is the major component of LH1-RC and LH2 and constitutes a special pair of RC (Cao et al., 2022). *R. alkalitolerans* photosystem also contains the pigment spheroidene, which plays a very important role in photoprotection and energy transfer being the accessory light-harvesting pigment (Nagy et al., 2004). Carotenoids have been shown to scavenge the ROS to safeguard the photosystem complexes during the photooxidative stress as a result of increased light stress (Nagy et al., 2004). The content of BChl *a* (Cohen-Bazire et al., 1957) is more in cultures grown in hpH conditions compared with hpH condition (Figure 1C), indicating that *R. alkalitolerans* accumulate more bacteriochlorophyll in hpH conditions. As carotenoids play an important role in scavenging ROS whereas BChl *a* majorly absorbs the energy, we calculated the carotenoid to BChl *a* ratio as a function of the state of the cell under photooxidative stress. Here, we found that the ratio has increased with an increase in light intensity, but it is more in npH than hpH (Supplementary Figure S1).

### Measurement of pH of culture media while harvesting

Here, we have plotted the pH difference between the npH-harvested culture and the blank and the hpH-harvested culture and its blank. Uninoculated culture medium was used as blank separately for each pH condition. The pH of culture in normal pH medium has increased as the light intensity increases, except for the culture grown at 250 μmol photons m^−2^s^−1^ (Figure 1D). In contrast, in high pH grown medium, the pH difference in harvested culture from that of the blank has subsequently decreased.

### Absorbance spectroscopy analysis

**Figure 2:**
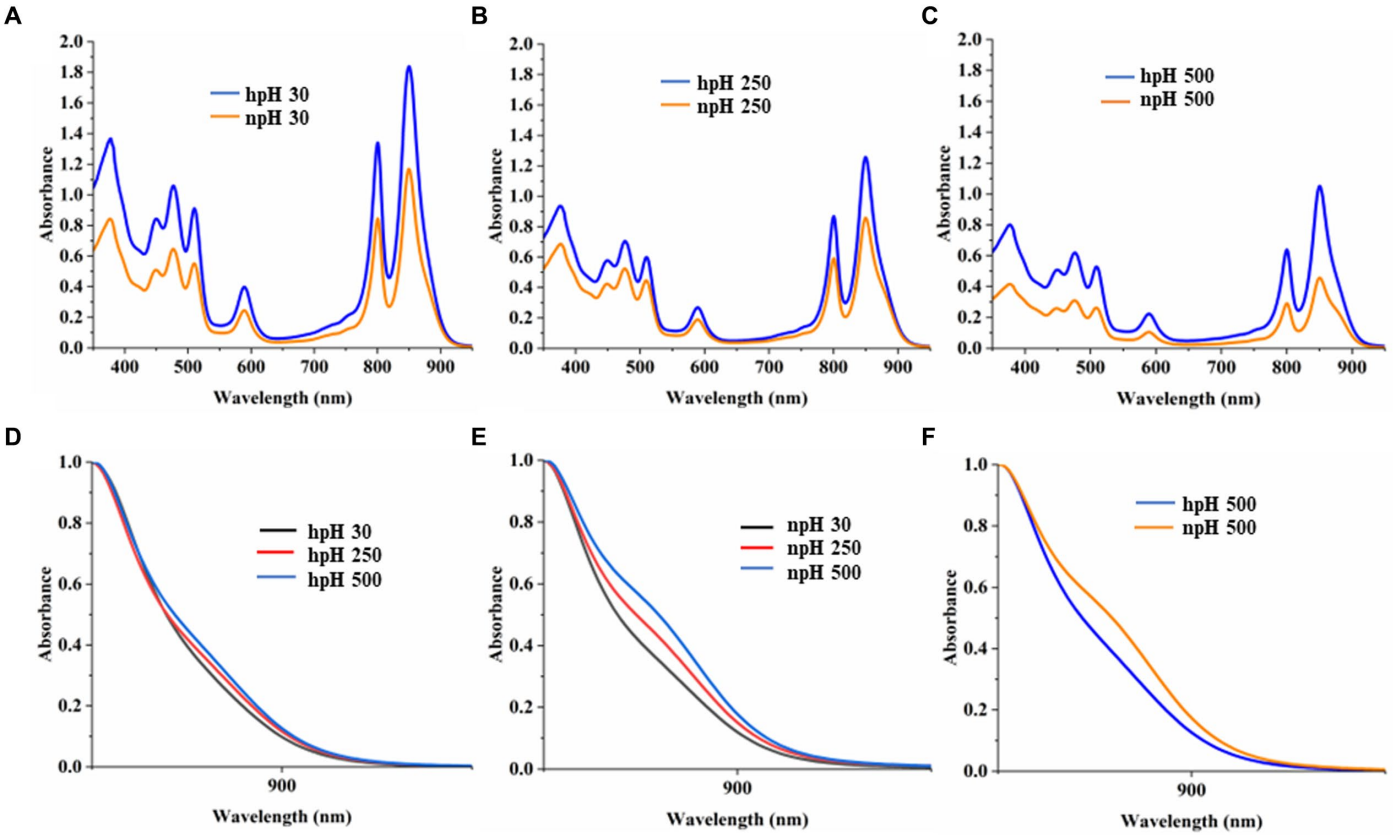
Comparative representation of absorbance spectrum of ICMs isolated from hpH-grown and npH-grown cells. ICMs were harvested from the interface of 15/40% discontinuous sucrose density gradient run overnight. **(A)** Absorbance spectra of npH and hpH from 30 μmol photons m^−2^ s^−1^. **(B)** Absorbance spectra of npH and hpH from 250 μmol photons m^−2^ s^−1^. **(C)** Absorbance spectra of npH and hpH from 500 μmol photons m^−2^ s^−1^. **(D)** Enlarged view of shoulder peak at 875 nm of hpH ICM absorbance. **(E)** Enlarged view of shoulder peak at 875 nm of npH ICM absorbance. **(F)** Enlarged comparative view of absorbance the npH and hpH ICM at 500 μmol photons m^−2^ s^−1^ light intensity.

A comparative study of the absorbance spectrum of ICMs shows that in hpH (pH 8.60 ± 0.05), the absorbance intensity is higher in all light conditions compared with npH (pH 6.80 ± 0.05) (Figures 2A–C). The absorbance spectrum is also an indication of photosystem integrity and stability of the photosystem in relation to given stress conditions (Niedzwiedzki et al., 2018). When increasing light intensity, the ICMs in both normal and high pH complexes are reduced, but in npH, the complexes are more sensitive than hpH. Absorbance data at equal protein concentration show that the spectrum intensity is comparatively higher in high pH conditions compared with normal pH conditions, indicating the role of high pH tolerance ability in photoprotection. The shoulder peak in the absorbance spectrum at 875 nm represents the RC-LH1 complex. In hpH its peak intensity is less compared to that of the npH ICMs which signifies that in npH condition the RC-LH1 has increased with an increase in light intensity (Figures 2D–F).

### Large pore blue native page and identification of protein subunit from selected protein complex

**Figure 3:**
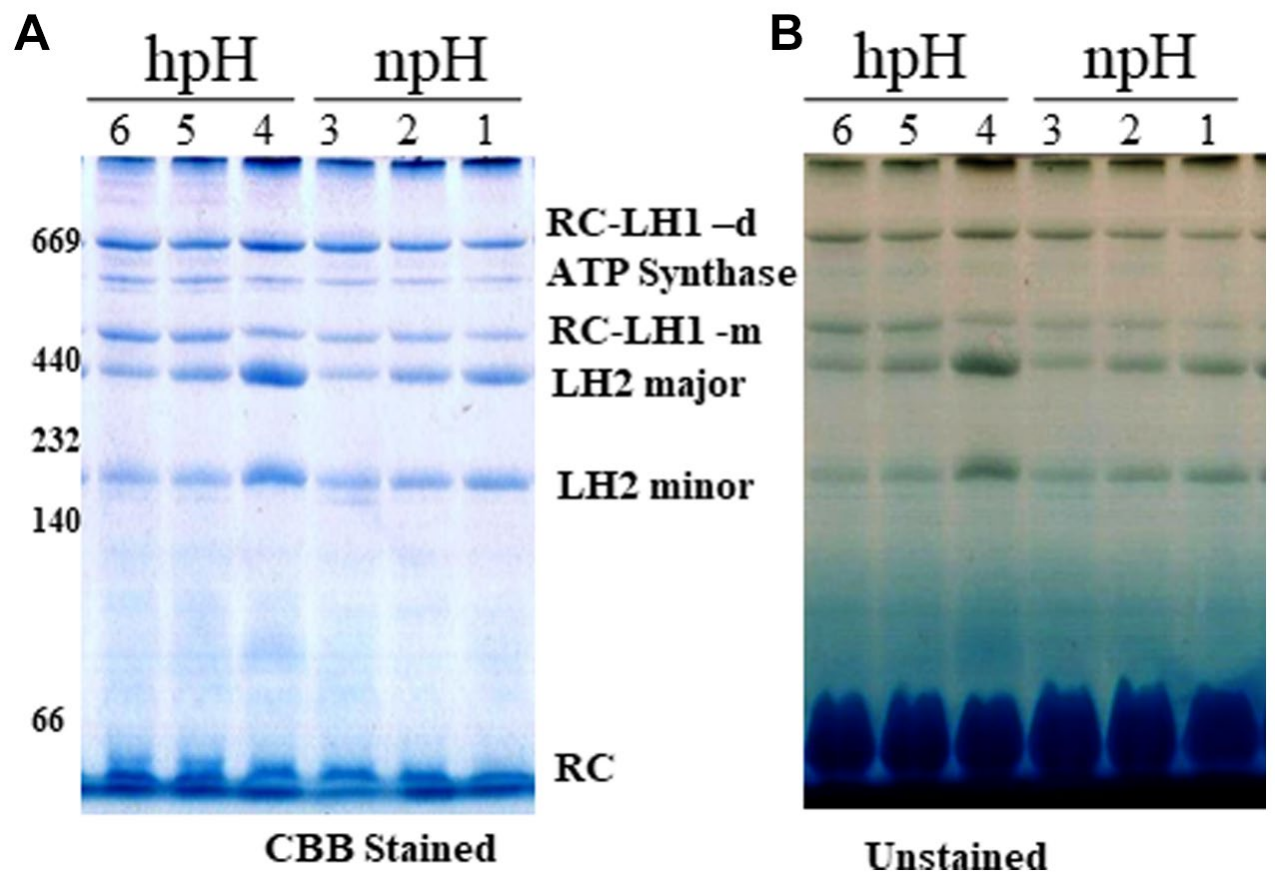
Large pore blue native page (LP-BN) of photosynthetic complexes. After centrifugation, supernatant was loaded in wells of native gradient gel of 3.5–12% by solubilization of intracytoplasmic membranes (ICMs) in 1% β-DM. Lane 1,2,3 represents the photosystem complexes from npH grown at light intensity of 30, 250 and 500 μmol photons m^−2^ s^−1^. Lane 4,5,6 represent photosystem complexes from hpH grown at a light intensity of 30,250 and 500 μmol photons m^−2^ s^−1^. At respective growth conditions, CBB stained with native marker lane **(A)** without stain **(B)**. Fifteen microgram of protein was loaded in each lane.

The blue native page separates the photosystem complexes without denaturing the photosynthetic pigment-protein complexes. Here, we found that as the light intensity increases, it affects the photosystem protein complex stability. A decrease is observed in the case of RC-LH1 dimer, although at 500 μmol photons m^−2^s^−1^, it is increased a little, and an increase is observed in RC-LH1 monomer in hpH condition which could be because of the conversion of dimer to monomer. In contrast, in npH-grown cells, it is the inverse of the hpH, wherein both dimeric and monomeric reaction centers have increased with the increase in light intensity (Figure 3; Supplementary Figures S2A,C). This could be an interesting phenomenon to pave the way to understanding the real reason for dimer monomer interconversion. The LH2 major and minor both have decreased with light intensity, but the extent is less in hpH conditions than in npH conditions (Figure 3; Supplementary Figures S2D,E). Even in normal growth conditions, the alkaline pH shows a stronger band of photosystem protein complexes than the normal pH. The protein complex separation pattern is in accordance with the previous study (Mothersole et al., 2016).

After staining the gel with colloidal Coomassie brilliant blue G -250, a non-pigmented band appeared below RC-LH1 dimeric band is enhanced with an increase in light intensity in both npH and hpH conditions (Supplementary Figure S2B). To identify this protein complex band, second dimension of the LP-BN strip was performed. The upper subunit protein bands were identified by mass spectrometry. Out of these, four protein bands were identified to be ATP synthase α (55.12 kDa), β (50.45 kDa), γ (31.2 kDa), and δ (19.34 kDa) (Supplementary Figure S3). The protein complex is found to be as ATP synthase complex with relatively increased expression with an increase in light intensity, and its expression is more in hpH conditions compared with npH conditions.

Furthermore, SDS-PAGE of ICMs from each condition shows that the protein abundance of photosystem complex proteins is higher in hpH than that in npH (Supplementary Figure S2F).

### Sucrose density gradient sedimentation and absorbance spectra of fractions from npH

**Figure 4:**
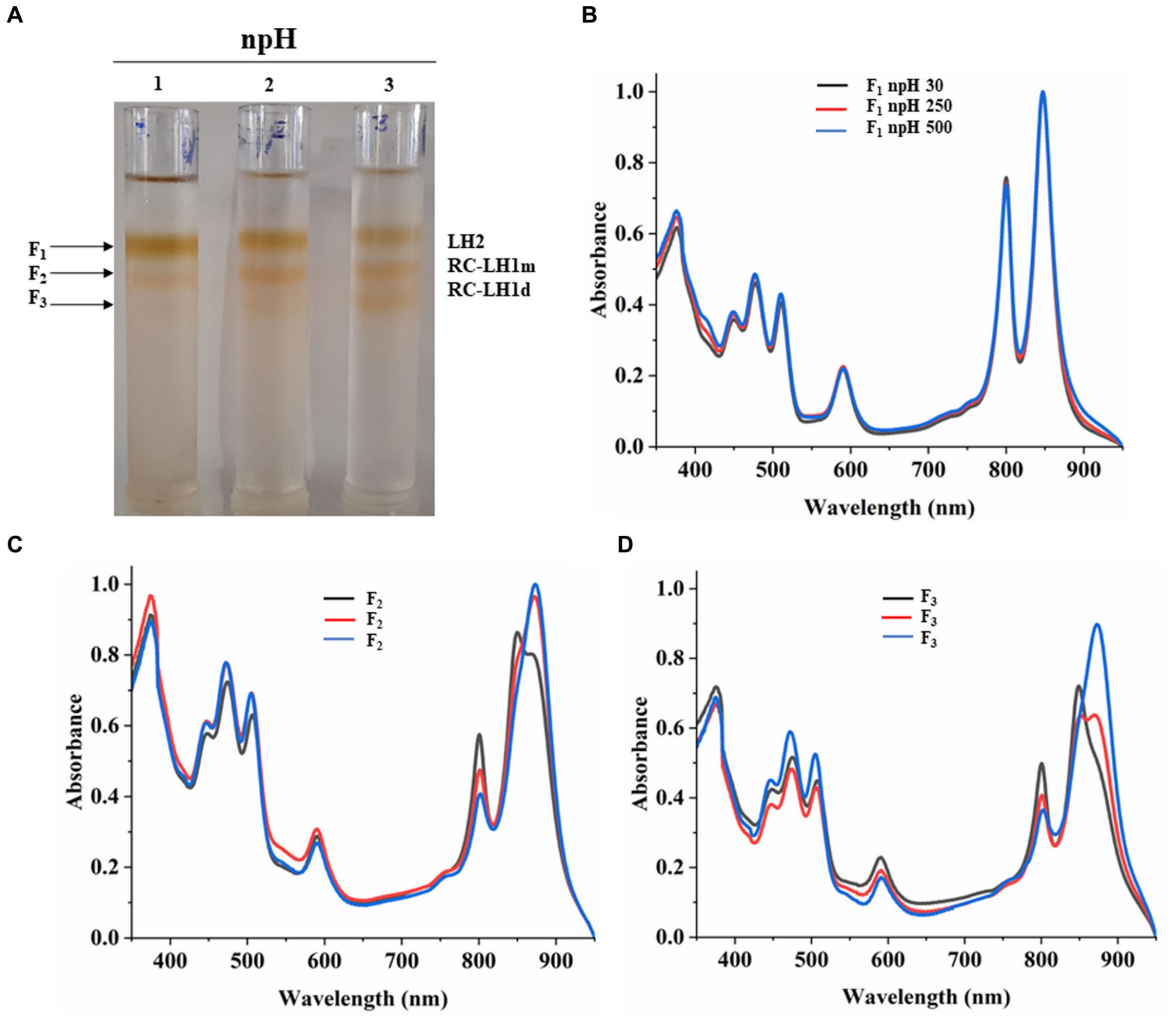
Sucrose density gradient of ICMs solubilized in 1% β-DM from npH. Five steps gradient of sucrose 20, 21.3, 22.5, and 23.8%, 25% (w/w) were made in 20 mM HEPES (pH 7.5) and 0.03% β-DM. Fractions were carefully harvested and their absorbance spectra was measured. 1,2,3 represent samples from light intensity of 30,250 and 500 μmol photons m^−2^ s^−1^, respectively **(A)**. Absorbance spectra of all three-fractions obtained F1 (LH2) **(B)**, F2 (RC-LH1m) **(C)**, F3(RC-LH1d) **(D)** in all three light conditions of 30, 250 and 500 μmol photons m^−2^ s^−1^.

Equal protein amounts of ICMs were solubilized in 1% β-DM loaded on the sucrose density gradient to separate the photosystem protein complexes of LH2 and RC-LH1 monomer and RC-LH1 dimer. Sucrose density gradient fractions were carefully collected, and their absorbance spectra were measured. SDG of npH condition shows that the F1 fraction, originating from light-harvesting antenna 2 (LH2), has decreased with increasing light intensity (Figures 4A,B; Supplementary Figure S4A). The same has also been found in LP-BN (Figure 3; Supplementary Figures S2D,E), although LH2 minor could not be separated in SDG because of minor difference in molecular weight (Mothersole et al., 2016), whereas both the F2 and F3 fractions have increased with an increase in light intensity. F2 represents RC-LH1 monomer and F3 represents RC-LH1 dimer (Figures 4B,C; Supplementary Figures S4B,C). The same is visible in the absorbance spectroscopy of each fraction of SDG. With an increase in light intensity, the RC-LH1 monomer and dimer have increased as the shoulder peak at 875 nm has enhanced while increasing in light intensity (Figures 2D–F, 4C,D). The same result is also observed in the LP-BN PAGE of ICM in npH, where both monomeric and dimeric reaction centers have elevated with an increase in light intensity (Figures 3, 4; Supplementary Figures S2A,C).

### Sucrose density gradient sedimentation and absorbance spectra of fractions from hpH

**Figure 5:**
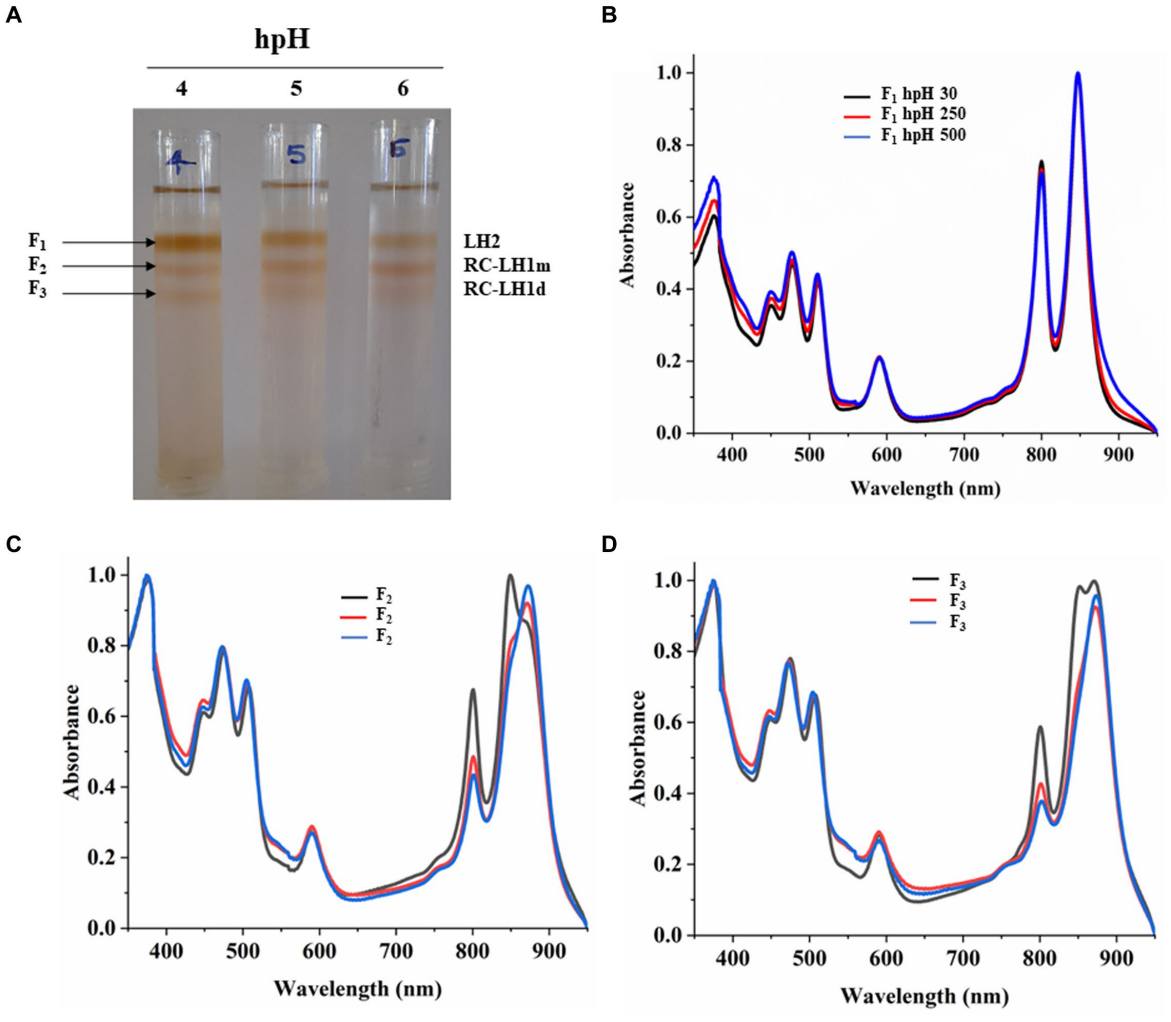
Sucrose density gradient of ICMs solubilized in 1% β-DM from hpH. Five steps gradient of sucrose 20, 21.3, 22.5, and 23.8%, 25% (w/w) were made in 20 mM HEPES (pH 7.5) and 0.03% β-DM. Fractions were carefully harvested and their absorbance spectra was measured. 4,5,6 represent samples from light intensity of 30,250 and 500 μmol photons m^−2^ s^−1^ **(A)**. Absorbance spectra of all the three-fractions obtained F1 (LH2) **(B)**, F2 (RC-LH1m) **(C)**, F3 (RC-LH1d) **(D)** and in all three light conditions of 30,250 and 500 μmol photons m^−2^ s^−1^.

Sucrose density gradient fractions were carefully collected, and their absorbance spectra were measured. SDG shows that the F1 fraction, which is for light harvesting (LH2), has decreased with increasing light intensity (Figures 5A,B; Supplementary Figure S4A). However, the F2 fraction, which is for the monomeric reaction center, has increased with light intensity. This could be because of the decrease in the F3 fraction which represented the dimeric reaction center complex, and its content was decreased (Figure 5A; Supplementary Figures S4B,C). It is also in agreement with Blue native PAGE (Figure 3; Supplementary Figures S2A,C). The absorbance spectroscopy of each fraction further confirms that the dimeric reaction center has converted to the monomeric reaction center (Figures 5C,D). However, a slight increase is observed in the dimeric reaction center (RC-LH1 d) at 500 μmol photons m^−2^s^−1^ of light intensity (Figure 5; Supplementary Figure S2A).

### Circular dichroism analysis

**Figure 6:**
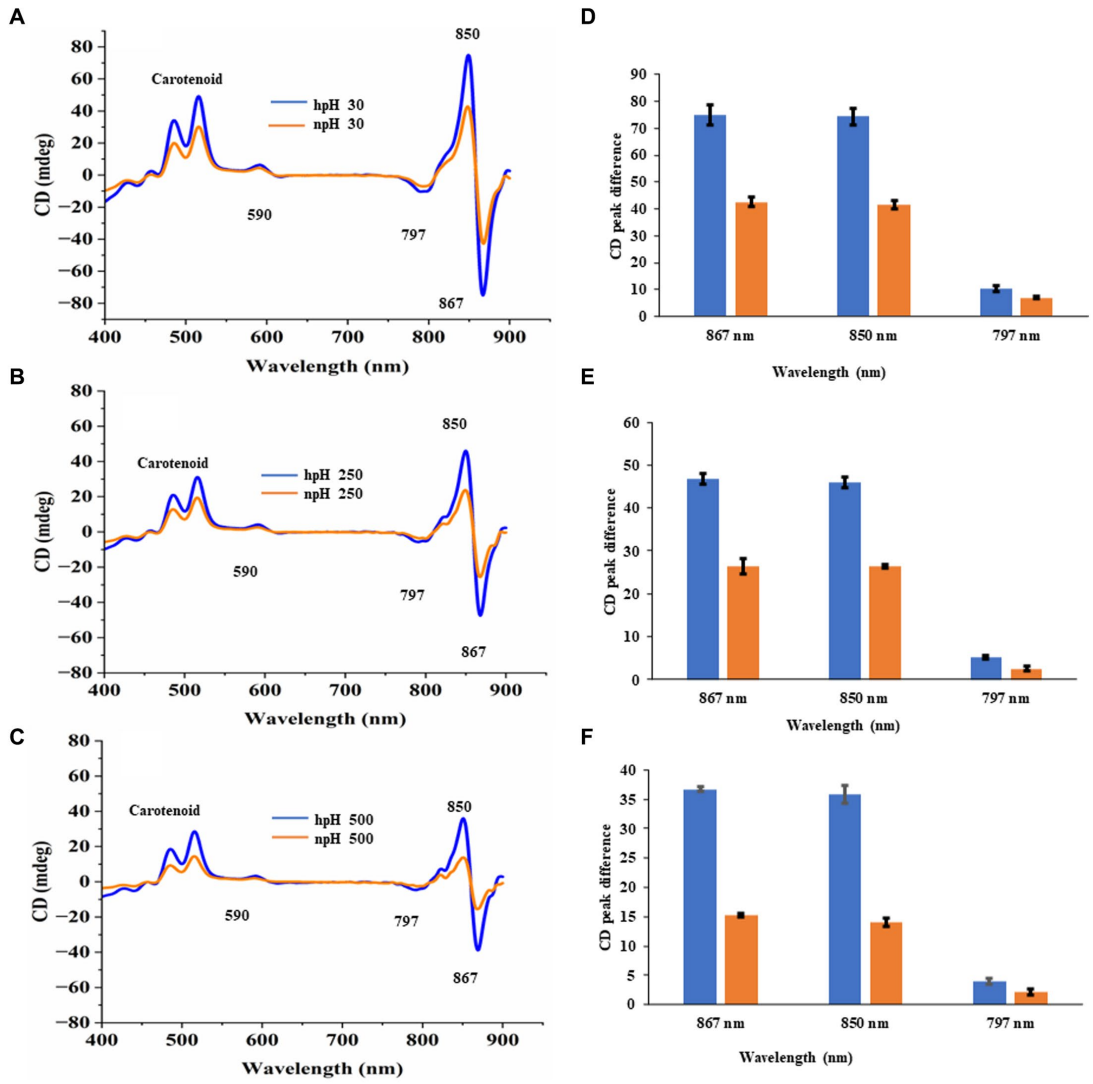
Comparative representation of circular dichroism of ICMs isolated from hpH and npH grown cells. CD spectra of ICMs (in 20 mM HEPES buffer with 10 mM MgCl_2_) at protein concentration of 50 μg/mL, were measured at room temperature using a Jasco J-1500 CD spectrometer. **(A)** Circular dichroism spectra of npH and hpH from 30 μmol photons m^−2^ s^−1^. **(B)** Circular dichroism spectra of npH and hpH from 250 μmol photons m^−2^ s^−1^. **(C)** Circular dichroism spectra of npH and hpH from 500 μmol photons m^−2^ s^−1^. Major peak intensity differences of circular dichroism spectra of ICMs from npH and hpH in all three light conditions **(D–F)**.

It is important to investigate the basic arrangement of the BChls in the complex in all the respective growth conditions. CD spectrum of ICMs from all these six conditions can provide detailed information about the organization and optically active pigments. In the CD spectrum, it is obvious that organizational pattern of pigment protein interaction remains the same. In contrast, the intensity of CD peaks is much stronger in hpH conditions than in npH-grown cells. The near-infrared (NIR) Q_Y_ region shows the doublet band with a strong positive band at 850 nm and a strong negative peak at 860 nm, pertaining to LH2 and LH1-RC (Tokita et al., 2011; Southall et al., 2018) (Figure 6). CD spectra have also shown the absorption peaks for the Q_x_ region at 590 nm and in the carotenoid region from 400 to 550 nm. Like the absorption spectra results, the CD spectra data also depict the changes in pigment-pigment/protein complexes during an increase in light intensity. These changes are much lesser in hpH than in npH, indicating that the complexes are stabilized in high pH. In addition, under high pH and high light, these complexes are relatively stable.

### Electrochromic shift analysis of whole cells

**Figure 7:**
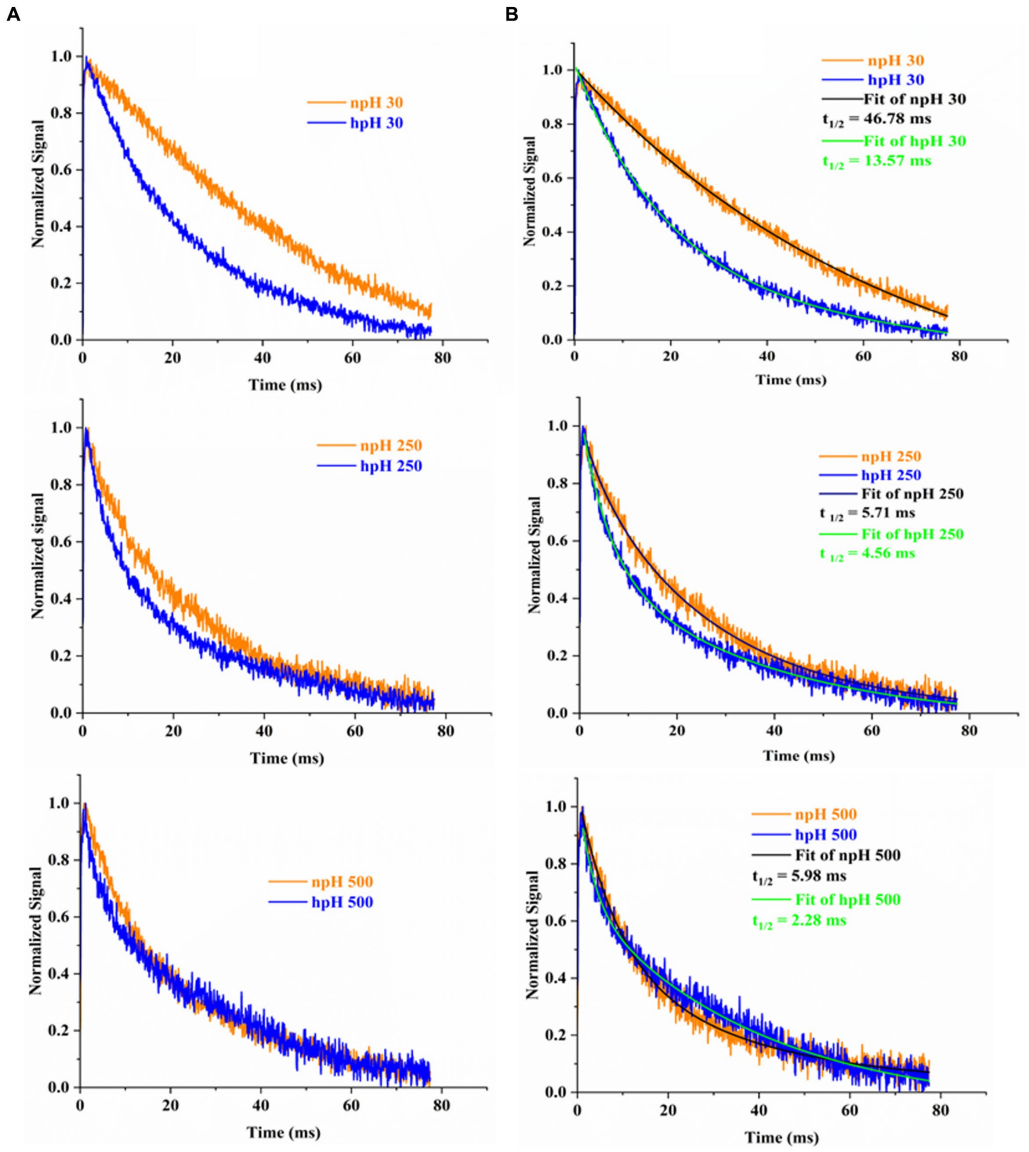
Flash-induced absorption changes in cells of *R. alakalitolerans* measured at 515 nm wavelength. Cells were adopted for 1 h in dark incubation before the measurement **(A)**. Calculation of t _1/2_ for each light intensity. Half-life of each decay kinetics was calculated by double fitting in origin software (Origin 2023). Half-life (t_1/2_) for npH and hpH at 30 μm photons m^−2^ s^−1^, t_1/2_ npH_30_ = 46.78 ms, t_1/2_ hpH _30_ = 13.57 ms. For 250 μmol photons m^−2^ s^−1^. npH and hpH t_1/2_ npH_250_ = 5.71 ms, t_1/2_ hpH_250_ = 4.56 ms and for 500 μmol photons m^−2^ s^−1^.in npH and hpH half-life were t_1/2_ npH_500_ = 5.98 ms, t_1/2_ hpH_500_ = 2.28 ms, respectively **(B)**.

**Figure 8:**
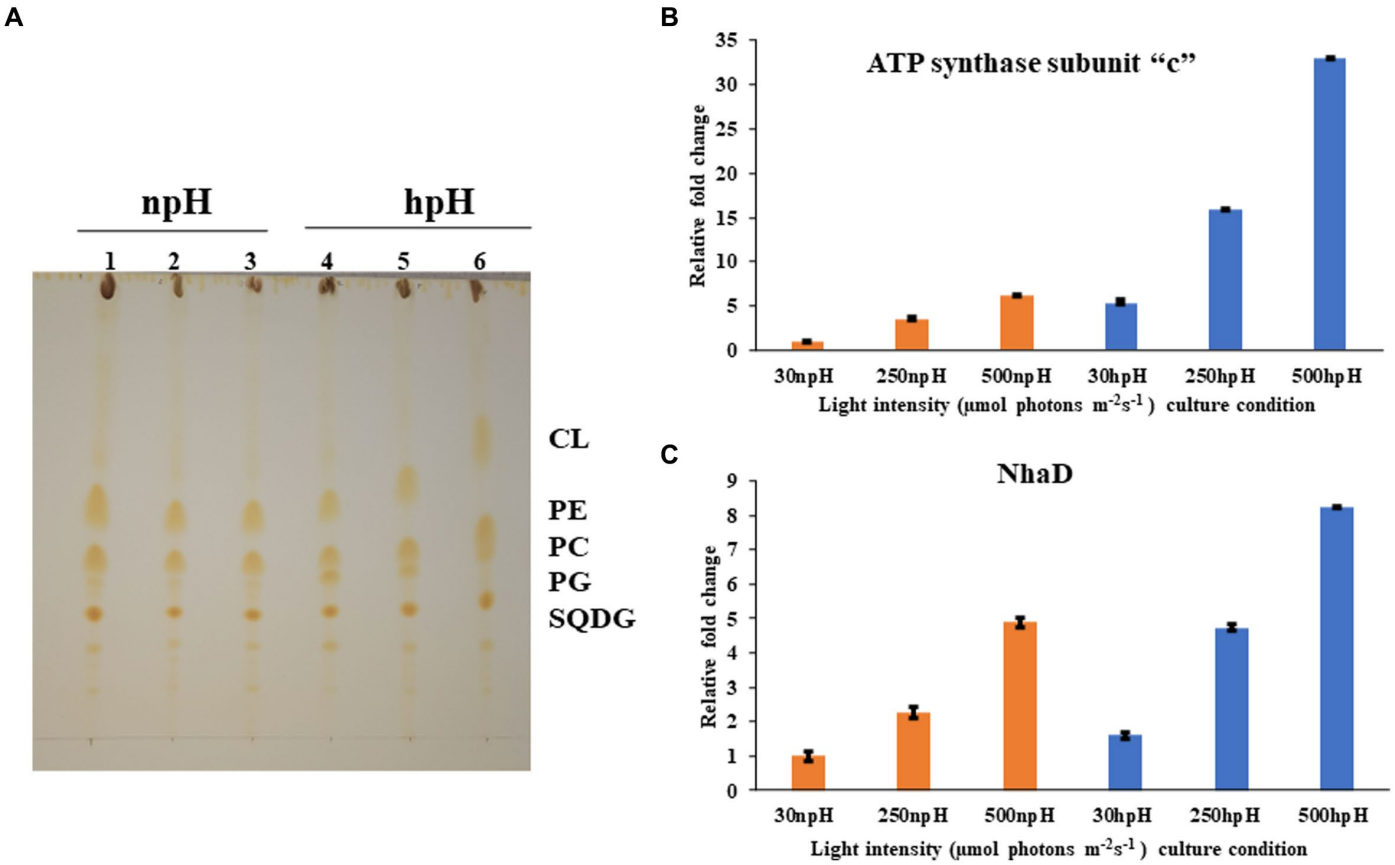
**(A)** Thin layer chromatographic separation of isolated polar lipids; Cardiolipin (CL) Phosphatidylethanolamine (PE) phosphatidylcholine (PC) phosphatidylglycerol (PG) and sufoquinovocyl diacylglycerol (SQDG). Membrane lipids were extracted in a mixture of methanol-chloroform-water (1:1:0.9, v/v) was added to 5 mg of lyophilized cells, separated by TLC on silica gel (20 × 20 cm, layer thickness 0.2 mm). and detected by iodine vapor. In npH (1,2,3 represent light intensity of 30,250,500 μmol photons m^−2^ s^−1^ in npH) and hpH (4,5,6 represent the light intensity of 30,250,500, in hpH μmol photons m^−2^ s^−1^). **(B)** q-PCR of subunit -c of ATPase in npH and hpH at light intensity of 30,250 and 500 μmol photons m^−2^ s^−1^. **(C)** q-PCR of sodium proton antiporter NhaD in npH and hpH at light intensity of 30,250 and 500 μmol photons m^−2^ s^−1^.

P515 measurement measures chromatophore lumen acidification in relation to ATPase activity by measuring flash-induced relaxation kinetics of carotenoids. Figure 7 shows the electrochromic shift measurement by P515 signal measurement of intact *R. alkalitolerans* cells grown in different conditions. The relaxation of the P515 normalized signal is also an indicator of ATPase activity. In this study, in high pH conditions, the relaxation is quite faster than that of the npH cells and has enhanced with an increase in light intensity (Figures 7A,B). Although in npH cells, the relaxation time has decreased, it is comparatively lesser than that of the hpH cells. The qPCR of the “c” subunit of ATPase expression level has also enhanced with an increase in light intensity which is more in hpH-grown cells (Figure 8B). It further confirms from LP-BN-2D and protein identification that the level of ATP synthase is higher in hpH-grown cells (Supplementary Figure S2B). This helps in less acidification in the chromatophore lumen and less detrimental effect on photosystem complexes.

### Quantitative real-time PCR and polar lipids

To assess the transcript level of the ATPase and the antiporter NhaD, qPCR was performed for subunit “c” of ATPase and NhaD. Both proteins play a crucial role in the dissipation of the proton gradient. NhaD has been shown to play a significant role in the homeostasis of bacterial cells when shifted in culture media of different alkaline conditions (Padan et al., 2005). ATP synthase also plays a very similar role to that of the NhaD, but it utilizes ADP (adenosine diphosphate and inorganic phosphate) to make ATP, which is used as an energy source in various metabolic processes and during photosynthesis by making the use of the proton gradient made in the cell. In this result, it is evident that the transcript level of both genes has increased significantly in hpH-grown cells compared with that of the npH-grown cells (Figures 8B,C). Moreover, expression levels of both genes have increased in response to an increase in light intensity.

Thin layer chromatography of polar lipids isolated from complete cells shows that this bacterium has sufoquinovocyl diacylglycerol (SQDG), phosphatidylcholine (PC), phosphatidylglycerol (PG), phosphatidylethanolamine (PE), and cardiolipin (CL) as major polar lipids, making lipid bilayer (Figure 8A). After analyzing the separation of lipids, the hpH-grown cells have a slightly higher amount of membrane lipids (Supplementary Figure S5). Out of this, phosphatidylcholine (PC) is present in higher amounts in hpH than in npH in all light intensities.

### Transmission electron microscopy

**Figure 9:**
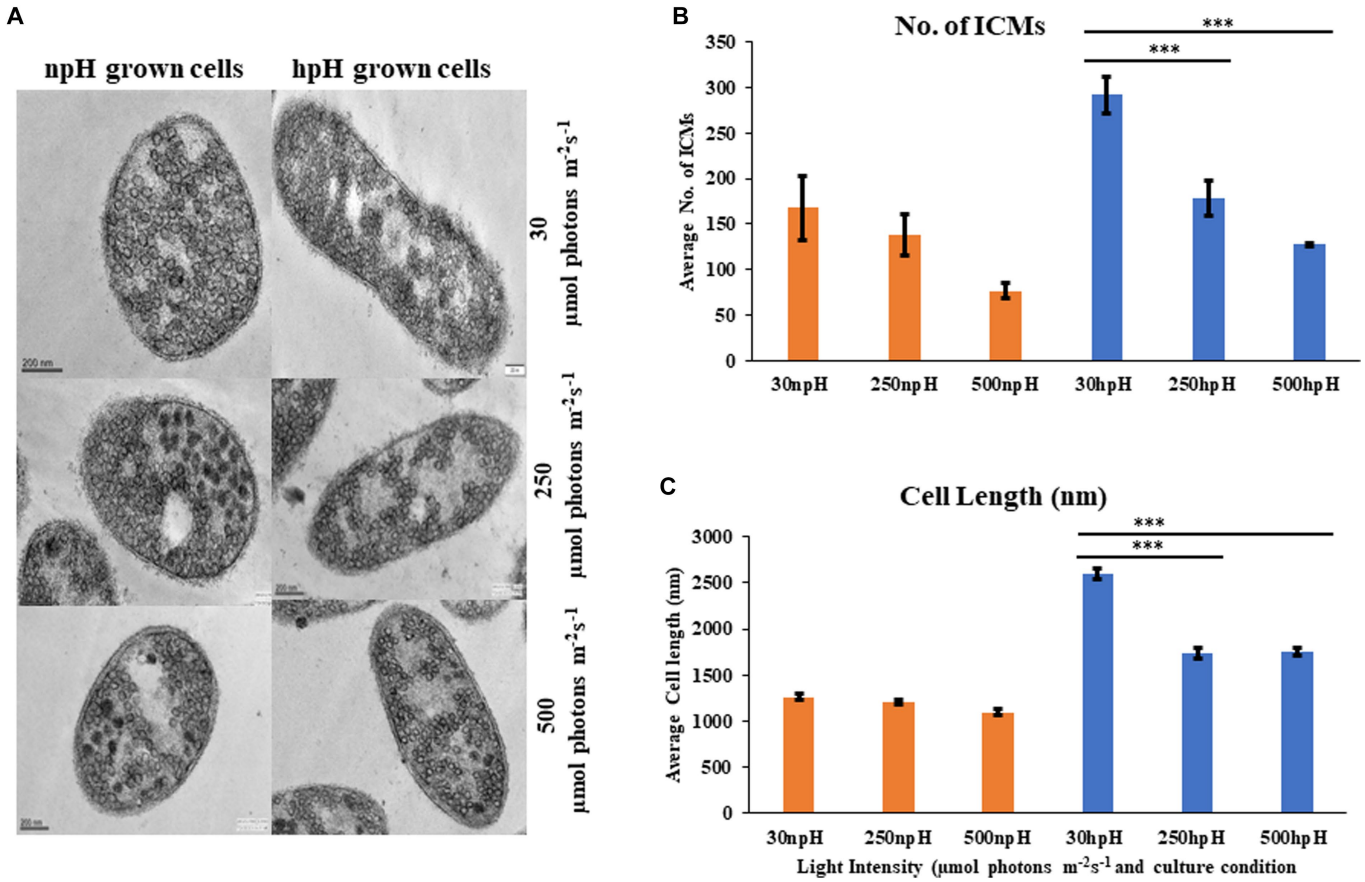
**(A)** Transmission electron micrograph of thin sections of *R. alkalitolerans* cells in various light intensities in npH and hpH culture condition. **(B)** Number of chromatophore/ICMs from npH and hpH condition at light intensity of 30, 250 and 500 μmol photons m^−2^ s^−1^. **(C)** Measurement of cell length (nm as imaged in the TEM instrument) in each growth condition from TEM images by ImageJ software (*n* = 3 cells were used). Asterisks indicated the level of significance of hight light (treated) versus control (normal light) with *p*-value style: GP: 0.1234 (ns), 0.0332(*), 0.0021(**), 0.0002 (***), and <0.0001 (****) respectively.

Electron micrograph of ICMs from *R. alkalitolerans* cells in all three light conditions in relation to culture condition npH shows that high light of 250 and 500 μmol photons m^−2^s^−1^ has drastically affected the ICMs compared with that of the hpH-grown cells. ImageJ analysis of TEM images from 250 to 500 μmol photons m^−2^s^−1^ of light intensity shows that npH-grown cells are much more affected than that of hpH-grown cells, and the number of ICMs has also decreased (Figures 9A,B). Another phenomenon is also apparent in hpH-grown cells that the cell size is increased (Figure 9C). In previous studies, in *E. coli,* cells grown in alkaline conditions were increased in size and length compared with cells grown at neutral pH (Mueller et al., 2020). In our study, the cell size has increased in high pH grown cells compared with that of the npH-grown cells. This property of elongation in cell size is more visible in normal light, whereas with an increase in light intensity, the cell size has decreased more in npH but in hpH, it remained almost the same at 250 and 500 μmol photons m^−2^s^−1^ of light intensity (Figures 9A,C).

### Estimation of total ROS and quantification of SOD

**Figure 10:**
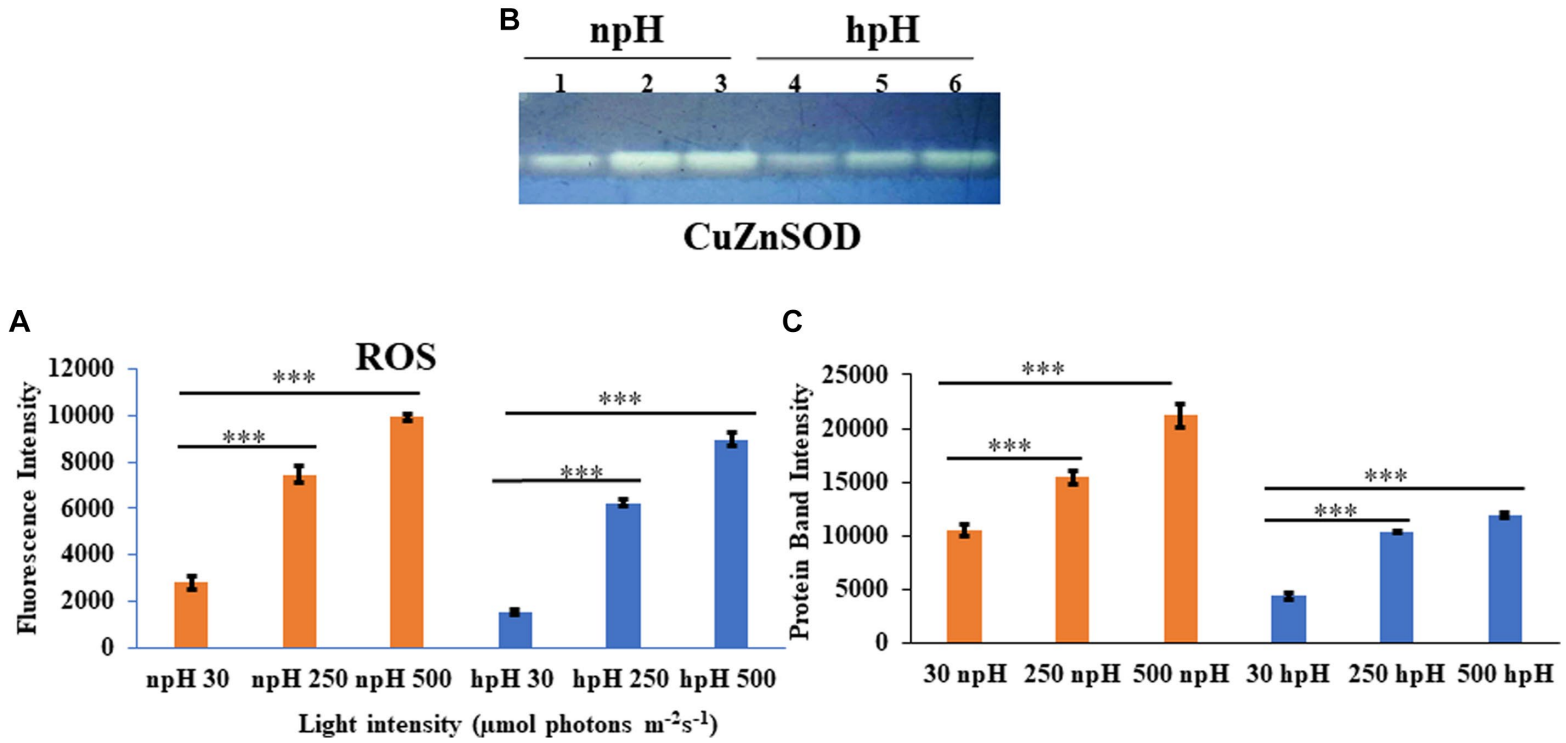
**(A)** Estimation of total reaction oxygen species (ROS) in cells grown in npH and hpH conditions. Cells from all the conditions were collected in logarithmic phase, and fluorescence intensity was measured at the excitation wavelength of 485 nm and emission at 530 nm. **(B)** Native polyacrylamide gel to detect level of Superoxide dismutase activity in npH and hpH conditions. After sonication lysate was centrifuged and supernatant was taken to estimate the protein concentration. Twenty microgram of protein was load in native gel to separate the protein. SOD bands were visualized after staining the gel in staining solution as described in materials and methods. Lane 1,2,3 represent samples from npH grown at 30,250 and 500 μmol photons m^−2^ s^−1^. Likewise lane 4,5,6 sequentially represent sample from hpH grown at 30,250 and 500 μmol photons m^−2^ s^−1^. **(C)** ImageJ quantification SOD bands from figure **(B)**. Asterisks indicated the level of significance of hight light (treated) versus control (normal light) with *p*-value style: GP: 0.1234 (ns), 0.0332(*), 0.0021(**), 0.0002 (***), and <0.0001 (****) respectively.

To measure the stress level in the cell in all conditions, the total ROS was measured by H_2_DCFDA fluorescent dye (Devadasu et al., 2021). ROS level has enhanced with an increase in light intensity in both npH and hpH conditions. It was found that the level of ROS is relatively high in npH-grown cells in all three light conditions compared with that in hpH condition (Figure 10A). To further investigate the level of superoxide dismutase expressed in the cell, the after-sonication cell supernatant was used to quantify the level of SOD. Only one band of SOD appeared after staining the gel with a staining solution containing nitroblutetrazolium chloride (NBT) (Figures 10B,C). According to Kho et al. (2006), the CuZnSOD is expressed, and its expression level has enhanced with an increase in light intensity in both npH and hpH conditions, but it is relatively less in hpH conditions.

## Discussion

### Effect of high light on growth pattern, external milieu, bacteriochlorophyll, carotenoid, and absorbance spectra

*R. alkalitolerans* when grown in npH and hpH, in relation to an increase in light intensity, shows not much difference in terms of growth pattern at 250 and 500 μmol photons m^−2^s^−1^, except at 30 μmol photons m^−2^s^−1^, where it takes a bit longer to enter the log phase (Figure 1A). The generation time of the bacterium is almost similarly decreased in all light intensities in both npH and hpH conditions (Figure 1B). hpH-grown cells entered the stationary phase at a less optical density, which could be because of the nutrient exhaust relatively early in hpH as cells have to expend more energy in hpH to maintain the homeostatic balance (Padan et al., 2005), leading to the consumption of more nutrients. Bacteriochlorophyll *a* is the major photosynthetic pigment present in *R. alkalitolerans* along with carotenoids, such as spheroidene, which along with photosystem protein RC- L, M, H, and light-harvesting antenna LH1 and LH2 make the photosystem (Qian et al., 2005). The content of Bchl *a* has decreased with an increase in light intensity, but the relative content is high in hpH-grown cells. This could be because of the cell metabolism, where more ATP is required to maintain the homeostatic balance in hpH, leading to very little damage to the photosystem because of high light; on the contrary, the cells in npH comparatively do not require much ATP. We also measured the pH of culture medium as to what happens to the extracellular pH while harvesting the cells. We found that there is an increase in the pH of culture medium by 0.5 units in npH conditions, whereas in hpH, the pH of culture has decreased with an increase in light intensity (Figure 1D). This indicates that growing the culture at high pH leads to the expression of antiporters (Padan et al., 2005; Adams and Hunter, 2012) (Figure 8C). This helps in maintaining the cytoplasmic pH of the cell, and subsequently, photoprotection of photosystem protein complex integrity in hpH condition relatively more than in npH condition (Figure 4). It is already been reported that when bacteria are grown in an alkaline environment, extracellular pH decreases (Padan et al., 2005), whereas when grown in npH, the extracellular pH of the culture medium increases (Farrell and Finkel, 2003; Padan et al., 2005; Sánchez-Clemente et al., 2018). The less increase in pH of hpH-grown culture compared to blank indicated that the cells are efficiently able to maintain the homeostatic balance by acid production and by proton antiporters not letting it increase but decreasing (Padan et al., 2005), where protons produced might be coming from photosynthetic machinery because increased expression enhanced ATPase activity (Figures 7A,B) has been observed along with relatively stable photosynthetic supercomplexes in hpH (Figures 3–5). From the data, it must be found out that the expression of antiporters might help in the dissipation of excess proton gradient built during high light in hpH culture, which leads to photoprotection in high light compared with that of the high light npH-grown cells (Padan et al., 2005), which is evidenced in the further experiments.

Absorbance spectroscopy of photosystem complexes represents the intactness and openness of the photosystems. The absorbance spectra of the isolated intracytoplasmic membranes in alkaline (hpH) conditions exhibit a more stable photosystem with high absorbance intensity compared with npH (Figures 2A–C). It clearly shows less impact of light stress on the photosystem of bacteria in hpH than that in npH (Figure 2). It is evident from Figures 2A–C that the shoulder peak at 875 nm which represents the RC-LH1 complex, either monomer or dimer, increased with an increase in light intensity. Comparing with the high light of 500 μmol photons m^−2^s^−1^, it is evident that the peak at 875 nm is higher in npH conditions, which could represent either RC-LH1 dimer or RC-LH1 monomer (Figures 2D–F). It also provides evidence that per RC-LH1, the number of LH2 is less in npH than in hpH, harnessing more energy required for hpH tolerance.

### LP-BN PAGE, sucrose density gradient of β- DM solubilized ICMs, and circular dichroism show relatively stable photosystem protein complexes in hpH

LP**-**BN- PAGE separates the photosynthetic protein complexes in native conditions (Kügler et al., 1997; Southall et al., 2018). Five major photosystem protein complexes were identified in each condition when compared with native page of *R. sphaeroides* (Mothersole et al., 2016), which is phylogenetically very near to *R. alkalitolerans* (Gandham et al., 2018). LP-BN shows major changes in RC-LH1 dimeric and monomeric complexes and light-harvesting antenna LH2. RC-LH1 complexes have decreased in hpH, and monomeric RC-LH1 complexes increased, which could be because of the conversion of dimeric RC-LH1 to monomer (Figure 3; Supplementary Figures S2A,C). This pattern is reversed in npH-grown cells, where both the dimeric and monomeric reaction centers have increased with an increase in light intensity. The increase in the content of both monomeric and dimeric RC-LH1 complexes in npH condition could be an adoptive and local microenvironmental strategy (Chenchiliyan et al., 2016) to cope with the effect of high light. In addition, it might be the photoprotective mechanism as the membrane lipids (bilayer and non-bilayer forming) are relatively less in npH with effect of high light and lesser ATPase activity. On the contrary, in hpH, it is opposite of npH. This pattern of conversion could be attributed to three factors as follows: (1) The microenvironment of the photosynthetic protein super complex includes the membrane bilayer forming PC, PG, and SQDG and non-bilayer forming lipids CL and PE. These membrane lipids provide differing environments around the protein complexes as and when they are changing, depending upon the growth condition. This has been previously shown to increase the photosystem protein complex stability by interacting with primary and secondary quinones along with other reaction center proteins (Ng et al., 2011). As in hpH, the membrane lipids CL, PC, PG, and SQDG are in relatively high content (Figure 8A; Supplementary Figure S5), which can provide a stable environment to protein complexes that are opposite in npH conditions. (2) Along with the membrane lipid environment, the level of ICM lumen acidification is also important in relation to the ATPase activity, which acts as the dissipater of proton gradient made as a result of photosynthesis. As light intensity increases, photons are absorbed, and the chromatophore lumen becomes more acidified because of proton accumulation. The acidic luminal condition of chromatophore leads to protein instability (Järvi et al., 2013; Krishnan-Schmieden et al., 2021). The level of ATPase complex has increased with an increase in light intensity in all conditions, but it is relatively high in hpH condition, which comparatively dissipates more proton gradient in hpH, leading to photoprotection. Previous studies show that in plant, thylakoid also has been shown to affect the photosynthetic protein complexes by high light-induced lumen acidification, activating many other photoprotective gene expressions, such as PsbS (Liu and Last, 2017; Krishnan-Schmieden et al., 2021). (3) Moreover, relatively fast relaxation of the P515 signal in hpH conditions compared with npH conditions, less lumen acidification effect on the protein complexes. Although at 500 μmol photons m^−2^s^−1^ in hpH, the content of RC-LH1 dimer increased a little bit, where it might start making dimers again as stress level increased. The light-harvesting antenna LH2 major and minor have also been drastically affected (Figure 3; Supplementary Figures S2D,E, S4A) in npH compared with hpH, which further confirms less photoprotection in npH. The sucrose density gradient from npH and hpH again proves the same pattern of protein super-complexes (RC-LH1 d, RC-LH1 m, and LH2) as of LP-BN. F1 represents the LH2, F2 represents the RC-LH1 monomer, and F3 represents the RC-LH dimer (Figures 4, 5). From this data, we can conclude that the photosystem protein complexes, antenna (LH2 major and LH2 minor) and core (RC-LH1 monomer and dimer), are relatively more stable in hpH condition. It can also be said that in hpH, the cell requires more energy to maintain the homeostatic balance. Moreover, to harvest more energy, the antenna size (higher content of LH2 is observed) is more in hpH than that in npH conditions.

To investigate the interactions between the pigment and protein which also represents protein complex integrity, we performed CD of ICMs at room temperature (RT) from the visible region to the far-red region (400–900 nm) (Clayton and Clayton, 1981; Davis et al., 1995), wherein the doublet bands at 875–850 nm show a particular orientational interaction of BChl *a* with that of the scaffold proteins of LH1and LH2. However, the CD pattern in NIR shows almost the same interaction pattern in complexes in three different light conditions (Figures 6A–C). The CD signal is affected as the culture grows in higher light intensities. However, less reduction was observed in pigment-protein interaction from hpH-grown ICMs (Figures 6D–F). It has been shown in the studies that the position of the carotenoid bands and the bacteriochlorophyll carotenoid ratio affect the NIR-CD spectra (Georgakopoulou et al., 2006).

### ECS measurement, membrane lipids, qPCR of ATP “c,” and NhaD have increased activity and levels of expression in hpH

The electrochromic shift measurement (Feniouk et al., 2002; Yakovlev et al., 2022) measures the ATPase activity as an electronic absorbance transient of intrinsic carotenoid spheroidene (Tokita et al., 2011). Spheroidene is the only carotenoid present in *R. alkalitolerans* cells (Gandham et al., 2018). The electrochromic shift in carotenoid spheroidene and spheroidenone results from increased membrane potential and membrane energization due to more chromatophore lumen acidification and absorption of light (Feniouk et al., 2002). Change in the permanent dipole moment of carotenoids upon excitation, and because of change in the electronic interaction, caused alterations in the protein organization (Gottfried et al., 1991; Feniouk et al., 2002). The experiment was performed in whole cells in all growth conditions, which shows that relaxation of the P515 signal is very fast in hpH compared with npH, which signifies the role of ATPase in hpH and its more immediate activity (Figure 7). The half-time of relaxation kinetics has decreased with an increase in light intensity. Although the half-life at 250 μmol photons m^−2^s^−1^ is less than 500 μmol photons m^−2^s^−1^ (5.71 ms < 5.98 ms) in npH, in LP-BN quantification, protein band has sequentially increased, although it (half-life) has sequentially decreased in hpH. The reason could be at the activity level that it might have retarded a little, whereas protein content might remain the same. Further qPCR of subunit “c” level has also increased with an increase in light intensity (Figure 8B), which supports the ECS measurement results.

Membrane lipids play a very important role when it comes in membrane proteins. They are the ones that provide the microenvironment for the well-functioning of these membrane proteins (Ng et al., 2011). So, it is important to study the membrane lipids while investigating the role of photosystem protein complexes as membrane energization is an important part of photosynthesis when it houses the carotenoids and bacteriochlorophylls (Ng et al., 2011). Since SQDG, PC, PG, PE, and CL are proven to be involved in the stabilization of the reaction center, especially PC has been shown to fasten the electron transfer rates in charge recombination states of Q_A_ and Q_B_ (Ng et al., 2011) and mediate more efficient formation of ICMs (Kim et al., 2007). These membrane lipids from whole cells and membrane lipids from ICMs have relatively no significant difference and are present in the same ratio (De Leo et al., 2009). SQDG, PC, and CL have previously been shown to interact with bacterial pheophytin and quinone (Ng et al., 2011) and reaction center proteins to enhance the stability of photosystem protein supercomplexes (Cao et al., 2022). Even CL and PG have been shown to increase the electron transfer rate between quinones (Ng et al., 2011). A case study in *R. sphaeroides* focused on the role of PC in the stability of the B800-850 complex, playing an important role in the efficient formation of ICMs (Kim et al., 2007), and ICM formation also confirms the importance of this lipid in the current study. At the same time, CL also plays an essential role in photosystem complex stability (De Leo et al., 2009). Overall, the increased membrane lipid in hpH conditions provides a relatively stable microenvironment and, subsequently, provides photoprotection.

Apart from membrane lipids, proton antiporters present in the plasma membrane also utilize intracellular protons to maintain homeostatic balance to the cells in hpH (Padan et al., 2005). Comparatively increased level of sodium proton antiporter NhaD in the hpH condition shows that to maintain the homeostatic balance in hpH, the antiporter level has increased. As previously reported, the sodium/potassium/calcium proton antiporter expression increases when bacteria are shifted to an alkaline environment (Padan et al., 2005).

### Transmission electron microscopy of cells and ROS estimation show relatively less oxidative stress in hpH

From TEM data, it is visible that high light impacts not only the cell size but also the number of ICMs in both npH and hpH conditions (Figure 9). However, as the cell length is more in hpH, it is because of the less frequent cell division culminated by the hpH environment. As reported earlier in *Escherichia coli* (*E. coli*), cell length increases as it is subjected to growth in increasing pH media cultures (Mueller et al., 2020). The cell division proteins, FtsZ and FtsN, are not able to mobilize to the middle of the cell, and cell divisome is not formed as efficiently as in npH-grown cells (Mueller et al., 2020); inverse to hpH, cells in acidic medium have been reported to divide fast (Mueller et al., 2020). As evident from TEM in npH, ICM count is more affected than hpH, but how the photosynthetic supercomplexes are affected is not clear from TEM data. It is only evident from LP-BN, SDG data and by membrane lipid data, where they are more stable in hpH condition than in npH. As ICMs are present freely in the cytosol, it would get affected by the intracellular environment, and its protein complex stability would depend on both the ICM lumen environment and cytosolic environment. In continuation to the cellular environment, it is also necessary to measure the redox level of the cell. We quantified the total reactive oxygen species (ROS) to measure the stress inside the cell generated because of high light (Ziegelhoffer and Donohue, 2009). Here, it should be noted that cells are grown anaerobically; therefore, ROS generated is because of photooxidative stress. The level of reactive oxygen species has increased with an increase in light intensity in both npH and hpH conditions, but the relative content is less in hpH conditions, which indicates that cells in hpH are in relatively less photooxidative stress (Figure 10A). Further confirmation comes from superoxide dismutase activity in non-denaturing gel, where CuZnSOD level is also increased in relation to ROS level, which is more in npH than in hpH and enhanced with an increase in light intensity (Figures 10A–C). CuZnSOD has been shown to express in photoheterotrophic conditions, confirming that in *R. alkalitolerans,* there is a single type of SOD to express in photoheterotrophic conditions (Kho et al., 2006). In *R. sphaeroides* and *R. capsulatus,* more than one SOD enzyme has been found to be active, but in the case of *R. alkalitolerans,* only one CuZnSOD has been found when grown anaerobically. Overall, the ROS and SOD levels are relatively less in hpH condition, which shows that the cell is under less photooxidative stress in hpH (Figures 10A–C).

Overall, in this study, we tried to investigate the behavior of the photosynthetic apparatus of *R. alkalitolerans* by growing it in alkaline growth conditions as it was discovered in an alkaline pond in Gujrat, India. We found that *R. alkalitolerans* cells have comparatively more ICMs and stable photosystem complexes in hpH. The level of membrane lipids (bilayer and non-bilayer forming) has increased in hpH, which also confers the stability to photosystem complexes. Formation of a more dimeric RC-LH1 complex in npH with an increase in light intensity emphasizes the importance of its role in acclimatizing as ICM lumen gets more acidified, but ATPase activity is not as efficient as in hpH where dimeric RC-LH1 is converting to monomer along with the increased ATPase activity. The reason for dimer to monomer conversion or dimer formation is still elusive, but it is clear that lumen acidification might be playing an important role in dimer formation or its interconversion to monomer in the provided micro lipid environment in either npH or hpH. Overall, *R. alkalitolerans* cells could adapt to hpH conditions photoheterotrophically with a relatively stable photosystem and homeostatic balance of the intracellular environment. Relatively less photooxidative stress, enhanced activity of ATP synthase from ICMs and high expression of Na^+^/H^+^ (NhaD) antiporter in hpH in high light indicate the interdependency of homeostasis and photosynthetic machinery functioning in cellular homeostatic balance.

Furthermore, *R. alkalitolerans* opens up the window for an in-depth study into complex interrelation between cellular homeostasis and photosynthesis, applying this knowledge to generate alkali-tolerant algal and plant species to cope with harsh alkaline environments. Being a photosynthetic purple non-sulfur species, it can also emerge as a potential species for biohydrogen and bioplastic production. Furthermore, studies are required to explore the possibility of the potential applications.

## Data availability statement

The original contributions presented in the study are included in the article/Supplementary material, further inquiries can be directed to the corresponding authors.

## Author contributions

MZ: Conceptualization, Formal analysis, Investigation, Methodology, Validation, Writing – original draft, Writing – review & editing. SM: Formal analysis, Investigation, Methodology, Writing – review & editing. NM: Data curation, Formal analysis, Investigation, Methodology, Writing – review & editing. VC: Conceptualization, Data curation, Formal analysis, Resources, Supervision, Validation, Writing – review & editing. RS: Conceptualization, Data curation, Formal analysis, Funding acquisition, Project administration, Resources, Supervision, Validation, Writing – original draft, Writing – review & editing.

## Supplementary material

SUPPLEMENTARY FIGURE S1 Ratio of the content of carotenoid and bacteriochlorophyll a in npH and hpH culture condition at light intensity of 30, 250 and 500 µmol photons m^−2^s^−1^. Bacteriochlorophyll a and carotenoids were measured at 775 nm and 456 nm from whole after extracing the pigments in 7:2 ratio of acetone and methanol.

SUPPLEMENTARY FIGURE S2 ImageJ analysis of LP-BN protein complex bands to compare the relative protein complex band intensity in npH and hpH condition at 30 µmol photons m^−2^s^−1^, 250 µmol photons m^−2^s^−1^, & 500 µmol photons m^−2^s^−1^. **(A)** RC-LH1 d, **(B)** ATP synthase complex, **(C)** RC-LH1 m **(D)** LH2 major, **(E)** LH2 minor. **(F)** SDS-PAGE of intracytoplasmic membrane protein from all above-mentioned growth conditions to compare the photosynthetic membrane protein complex abundance (lanes 1&4, 2&5, 3&6 sequentially represent 30, 250, and 500 µmol photons m^−2^s^−1^ of light intensity). Each data point averages three replicates, and error bars are represented as ±SE. Asterisks indicated the le vel of significance of hight light (treated) vs control (normal light) with P value style: GP: 0.1234 (ns), 0.0332(*), 0.0021(**), 0.0002 (***), <0.0001 (****) respectively.

SUPPLEMENTARY FIGURE S3 The second dimension of the LP-BN strip for identification of ATP synthase complex subunits. Subunit proteins were excised and sent for identification **(A)** by Orbitrap Liquid Chromatography Mass Spectrometry (OHRLCMS). The sequence of peptides matched (red-coloured amino acid sequences) with respective full amino acid sequences **(B)**.

SUPPLEMENTARY FIGURE S4 Image-J analysis of sucrose density gradient fractions. **(A)** LH2 (fraction1: F1), **(B)** RC-LH1 m (fraction 2: F2) and (C) RC-LH1 d (fraction 3: F3). Fractions were obtained after 18 hrs of ultracentrifugation. Each data point averages three replicates, and error bars are represented as ±SE. Asterisks indicated the level of significance of high light (treated) vs control (normal light) with P value style: GP: 0.1234 (ns), 0.0332(*), 0.0021(**), 0.0002 (***), <0.0001 (****) respectively.

SUPPLEMENTARY FIGURE S5 ImageJ quantification of membrane lipid band from TLC plate. **(A)** Cardiolipin, **(B)** Phosphatidylethanolamine, **(C)** Phosphatidylcholine, **(D)** Phosphatidylglycerol, **(E)** Sulfoquinovosyldiacylglycerols. Each data point averages three replicates, and error bars are represented as ±SE.

SUPPLEMENTARY TABLE S1 Nucleotide sequence of forward and reverse primer used for RT-PCR study of recA, NhaD and ATP synthase subunit “c” genes.
