# Supplementary material for: Differential stability of bacterial photosynthetic apparatus of Rhodobacter alkalitolerans strain JA916T under alkaline and light environment
Source: Front Microbiol. 2024 Mar 14;15:1360650. doi: 10.3389/fmicb.2024.1360650 (PMC10977657; doi:10.3389/fmicb.2024.1360650)
Supplement: Supplementary file 1 [file Data_Sheet_1.zip › fmicb.2024.1360650/Data Sheet 1.pdf]

Supplementary Figure. S1

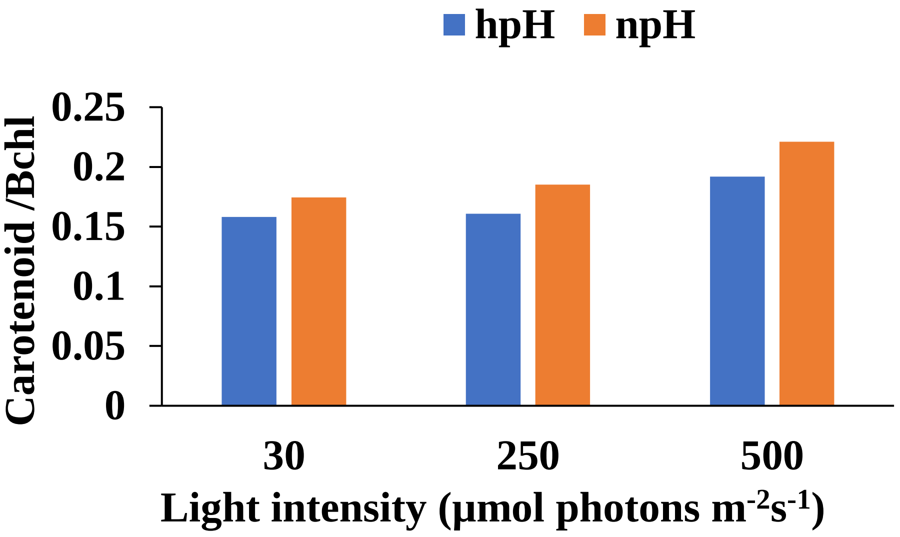

Supplementary Figure. S2

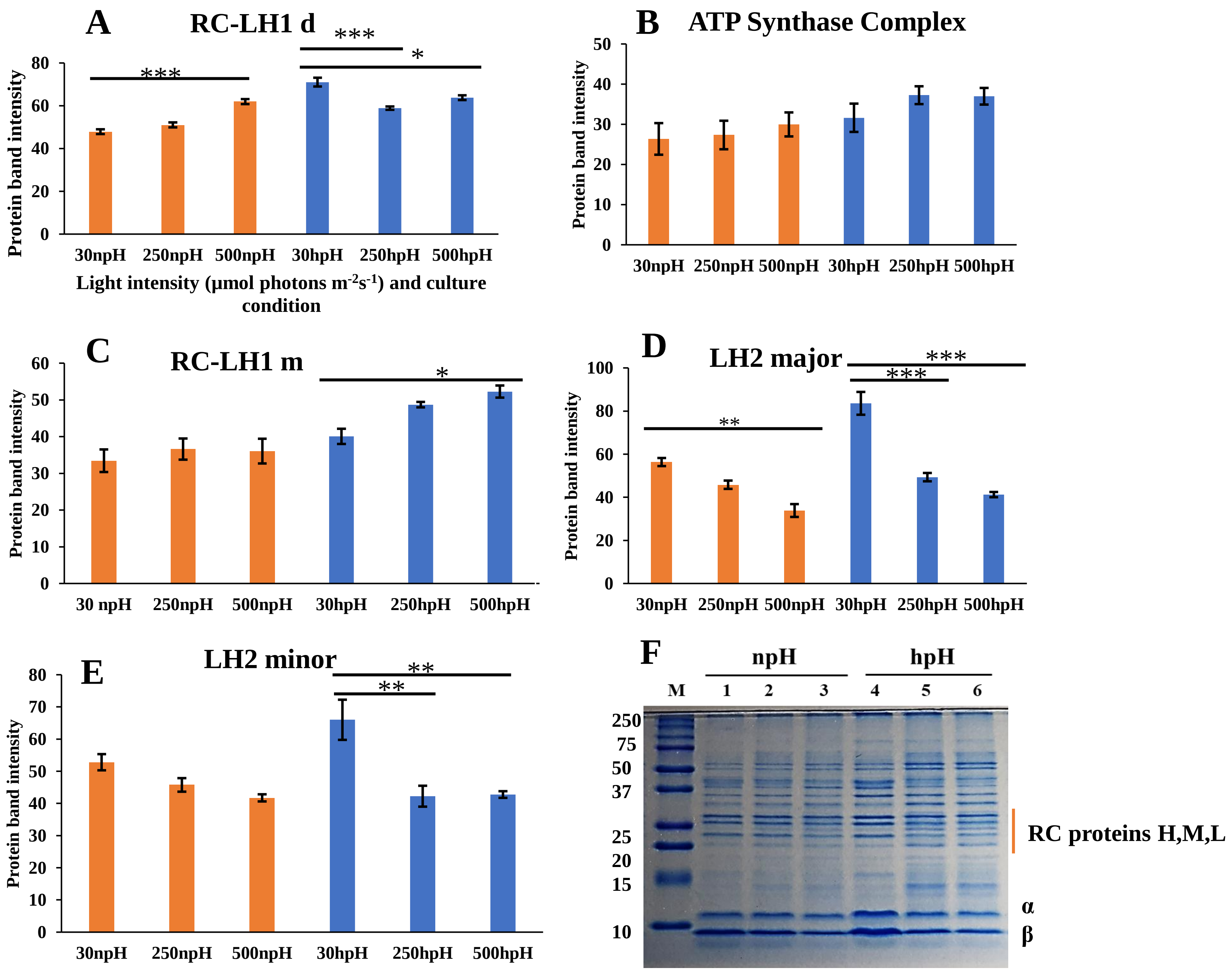

Supplementary Figure. S3

A

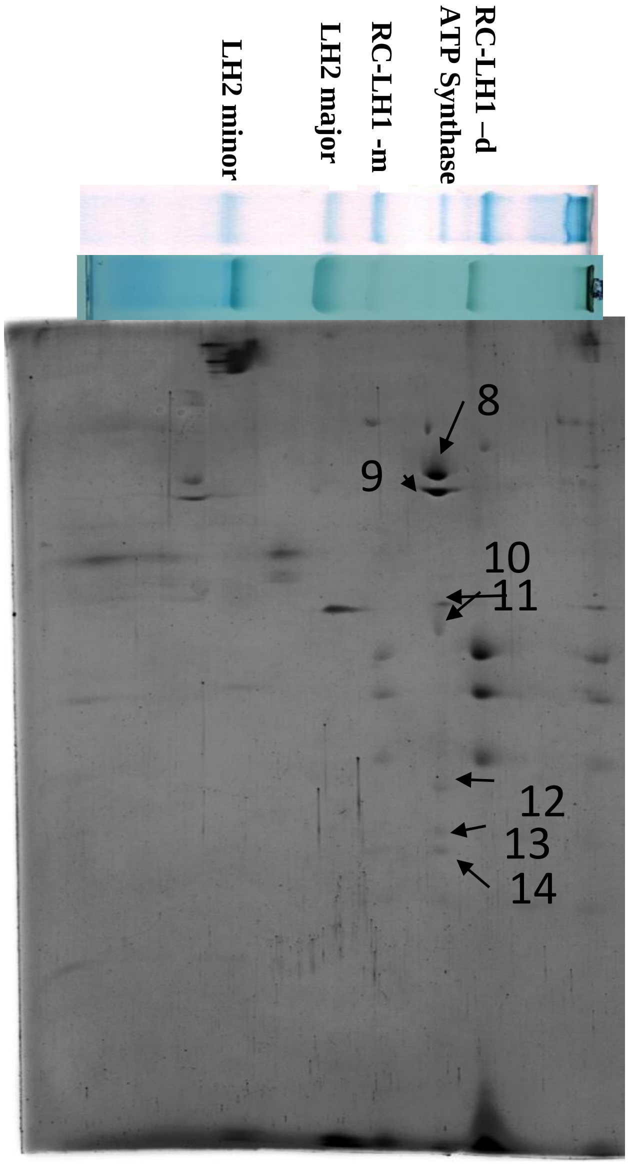

B

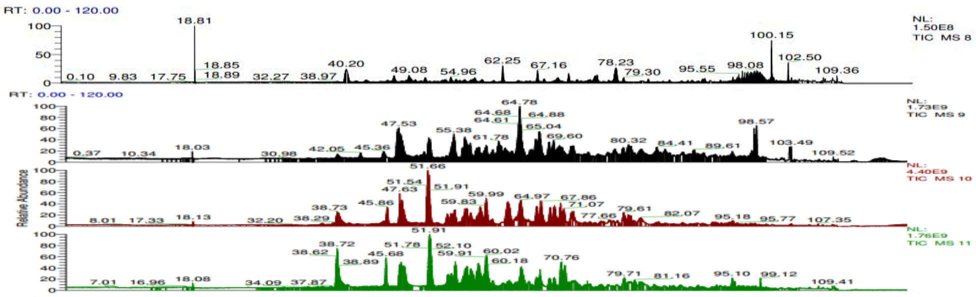

>sp|A4WUM9|ATPA_CERS5 **ATP synthase subunit alpha (protein band 8)**
MGIQAAEISAILKEQIKNFGQAAEVAEVGRVLSVGDGIARVHGLDNVQAGEMVEFPGGIR
GMALNLEVDNVGVVIFGDDRSIKEGDTVKRTKSIVDVPAGDALLGRVVDGLGNPIDGKG
PIAATERRVADVKAPGIIPRKGVHEPMATGLKSVDAMIPIGRGQRELIIGDRQTGKTAIAL
DTILNQKSYNEAAGDDESKKLYCIYVAIGQKRSTVAQLVKKLEETGAIAYTLVVAATASD
PAPMQFLAPYAATAMAEYFRDNGRHALIIYDDLSKQAVAYRQMSLLLRRPPGREAYPGD
VFYLHSRLLERSAKLNKEHGSGSLTALPIIETQGGDVSAFIPTNVISITDGQIFLETELFYQGI
RPAVNTGLSVSRVGSSAQTDAMKSVAGPVKLELAQYREMAAFAQFGSDLDAATQQLLN
RGARLTELMKQPQYAPLTNAEIVCVIFAGTKGYLDKVPVKDVGRWEQGLLKHLRTNAR
DLLADITNNDRKVKGELENKIRAALDTYAKDFA

>WP_011909162.1 MULTISPECIES: F0F1 **ATP synthase subunit beta (protein band 9)**
MATASQGKVTQVIGAVVDVQFDGGLPAILNALETVNNDKRLVLEVAQHLGENTVRTIA
MDATEGLVRGAPVTDLGGPISVPVGDATLGRILNVIGEPIDEKGPVSGDSTRAIHQPAPTF
AEQSTTSEILVTGIKVILLAPYSKGGKIGLFGGAGVGKTVLIMELINNIAKVHSGYSVFAGV
GERTREGNDLYHEMIDSGVIKIDNLSESKVALVYGQMNEPPGARARVALTGLTLAEQFR
DQSGTDVLFFVDNIFRFTQAGSEVSALLGRIPSAVGYQPTLATDMGALQERITSTKAGSITS
VQAIYVPADDLTDPAPATSFAHLDATTVLSRAISELGIYPAVDPLDSTSRILDPQIVGEEHY
NVARAVQGILQRYKSLQDIIAILGMDELSEEDKLTVARARKIQRFLSQPFDVAKVFTGSDG
VQVPLEKTIASFKAVVNGEYDHLPEAAFYMVGDIEDVIAKAQRLAAQAA

>sp|A4WUM8|ATPG_CERS5 **ATP synthase gamma (protein band 10)**

MPSLKDLKNRIGSVKNTRKITKAMQMVAAAKLRRAQEAAEAARPFAERMTAVMTGLA
GSVGSSESAPRLLAGTGSDKVQLLVVMTAERGLCGGFNSSIVRLARAHAAKLLAEGKTV
KILTVGKKGREQLRRDLGQHFIGHVDLSEVKRMGYPVAQGIARDVLDRFDKGEFDVATIF
FARFQSVISQIPTAQQVIPAVFEGEGEVNSLYDYEPSEEGVLADLLPRGVATQIFTALLENG
ASEQGARMSAMDNATRNAGDMINRLTIQYNRSRQAAITKELIEIISGAEAL

>tr|A0A7Z6QY53|A0A7Z6QY53_CERSP **ATP synthase subunit delta (protein band 11)**

MSEPASISSGIAARYAAAVFELAKDEGALPALEKDMDALGAAWSESADLRDLATSPVYA
REEQQKAIAAIAAKMGLSSLTANTLALMGSKRRLFVLPQMVADVQNRIATEKGEITAEVT
AAAPLSPEQAARLAATLKARAGKDVKLKTTVDESLIGGLVVKLGSSMIDTSVKARLAAL
QNAMKEVG

Supplementary Figure. S4

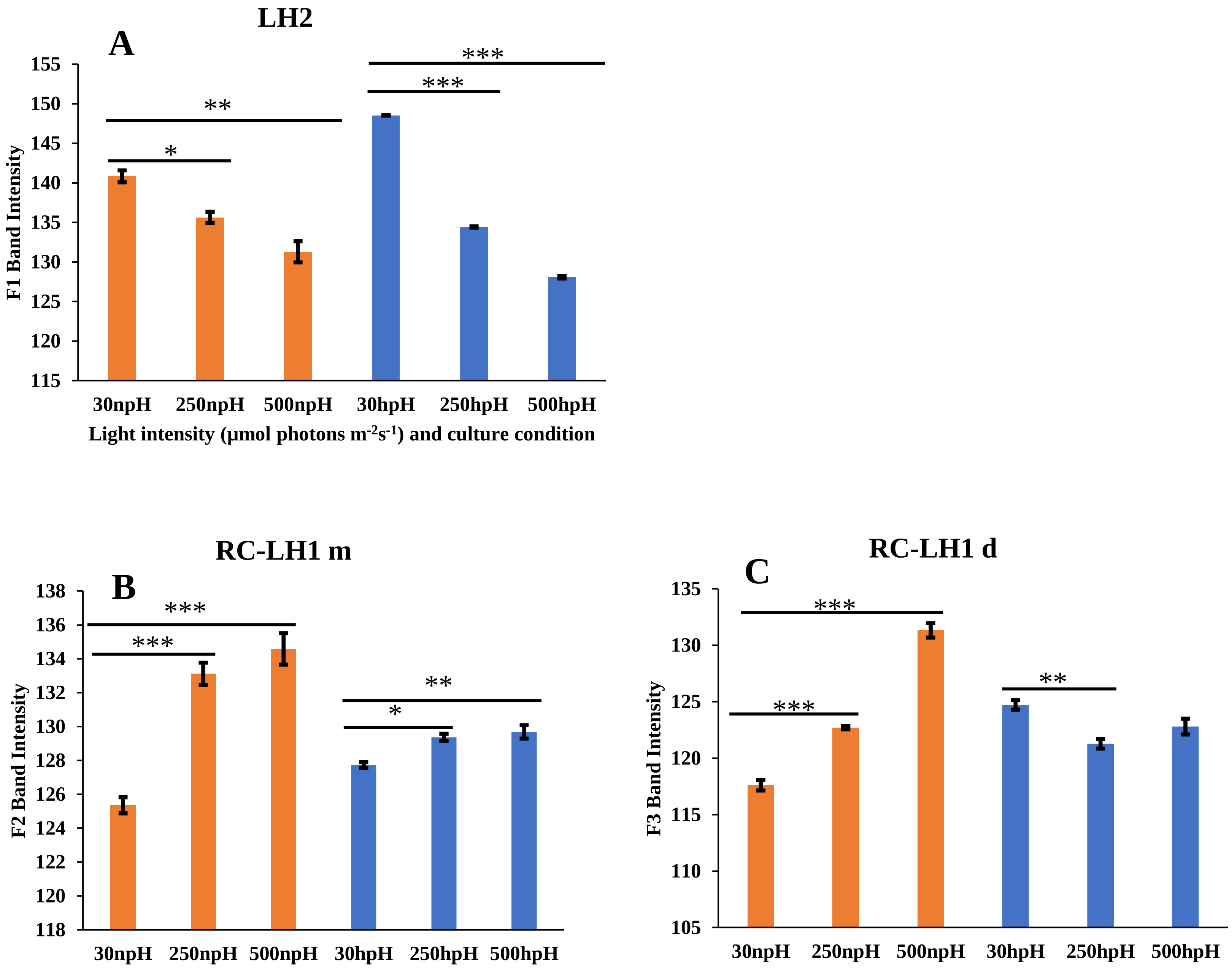

Supplementary Figure. S5

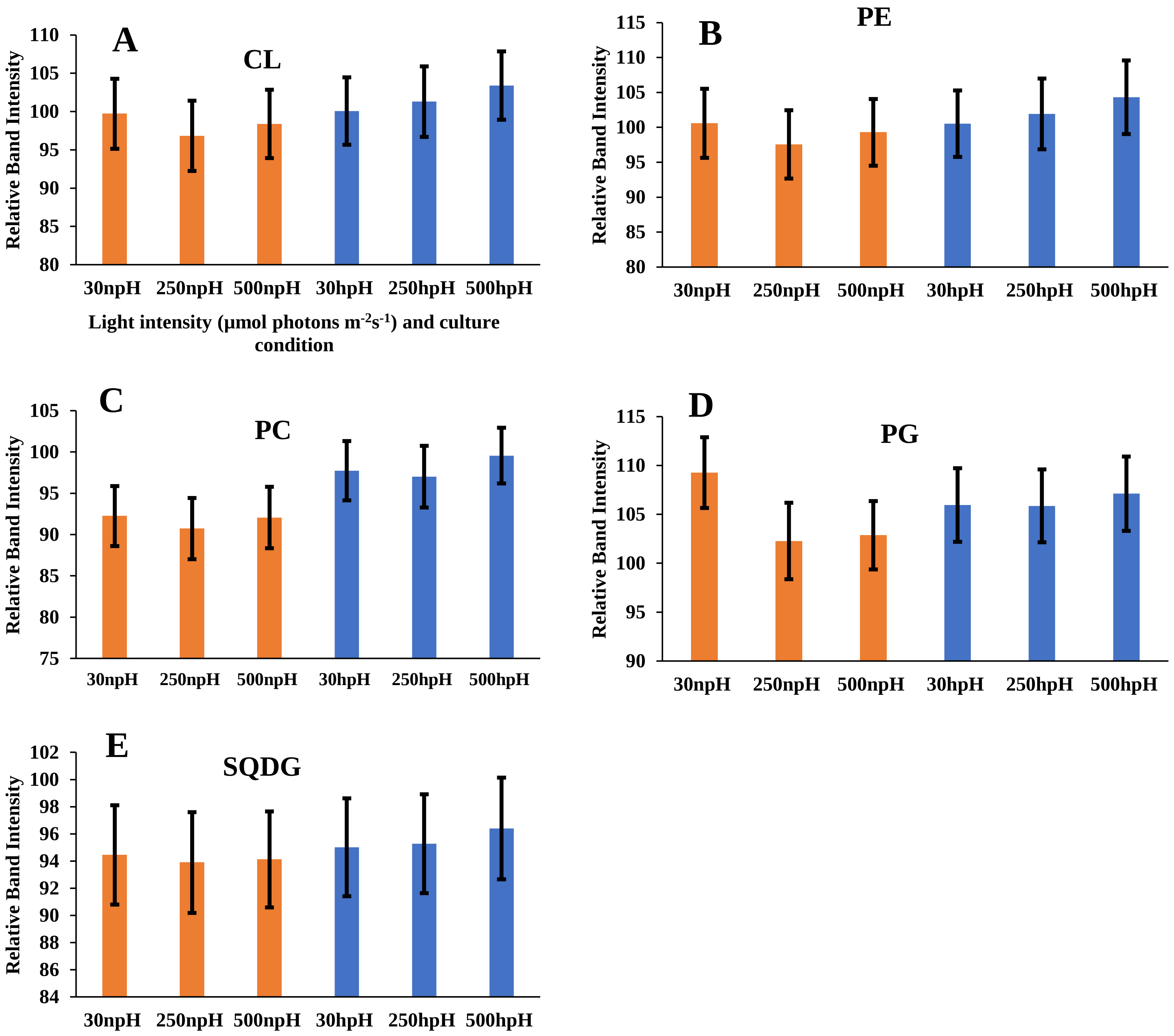

Supplementary Table S1.

Gene	Forward Primer	Reverse Primer
recA	5’ CTCGATCATGAAACTGGGG 3’	5’CACCCTTCTTCTGCTCTTC 3’
NhaD	5’ CATCATCGAGGAATATGGCG 3’	5’ GTCAGGTTGTCGAGGATG 3’
ATP synthase subunit “c”	5’ ATGGGCAAATTCATCGGC 3’	5’ CGATCAGGAACGAGAAGATG 3’
