# Supplementary material for: Differential stability of bacterial photosynthetic apparatus of Rhodobacter alkalitolerans strain JA916T under alkaline and light environment
Source: Front Microbiol. 2024 Mar 14;15:1360650. doi: 10.3389/fmicb.2024.1360650 (PMC10977657; doi:10.3389/fmicb.2024.1360650)
Supplement: Supplementary file 1 [file Data_Sheet_1.zip › fmicb.2024.1360650/Table 1.pdf]

Supplementary Table S1.

Gene	Forward Primer	Reverse Primer
**recA**	**5’ CTCGATCATGAAACTGGGG 3’**	**5’CACCCTTCTTCTGCTCTTC 3’**
**NhaD**	**5’ CATCATCGAGGAATATGGCG 3’**	**5’ GTCAGGTTGTCGAGGATG 3’**
**ATP synthase subunit “c”**	**5’ ATGGGCAAATTCATCGGC 3’**	**5’ CGATCAGGAACGAGAAGATG 3’**
