# Supplementary figures and images for: Differential stability of bacterial photosynthetic apparatus of Rhodobacter alkalitolerans strain JA916T under alkaline and light environment
Source: Front Microbiol. 2024 Mar 14;15:1360650. doi: 10.3389/fmicb.2024.1360650 (PMC10977657; doi:10.3389/fmicb.2024.1360650)

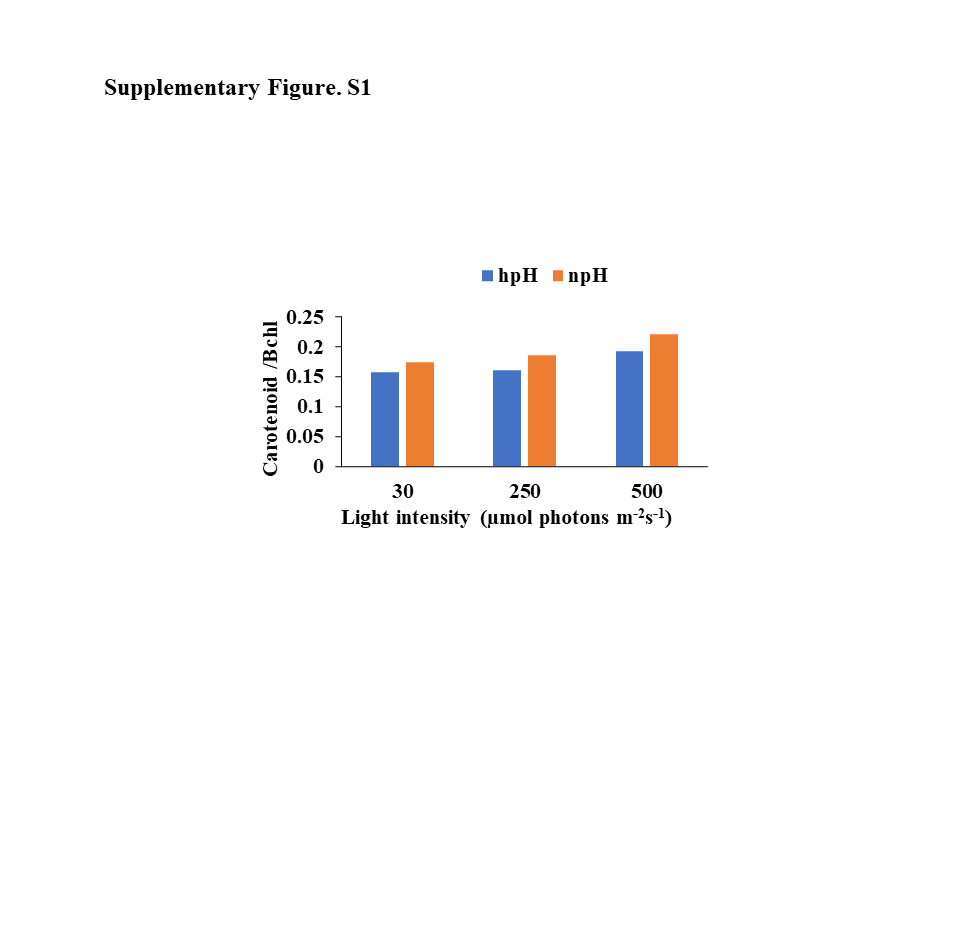

Supplement: Supplementary file 1 [file Data_Sheet_1.zip › fmicb.2024.1360650/Image 1.tif]

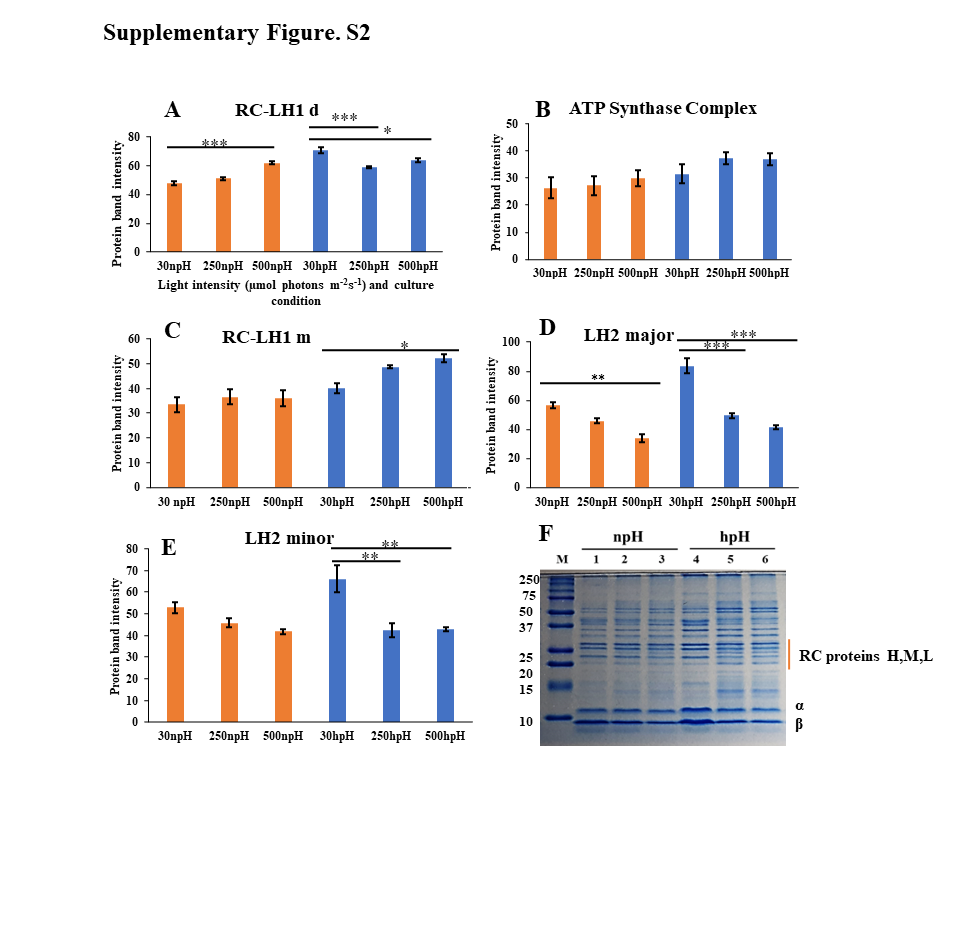

Supplement: Supplementary file 1 [file Data_Sheet_1.zip › fmicb.2024.1360650/Image 2.tif]

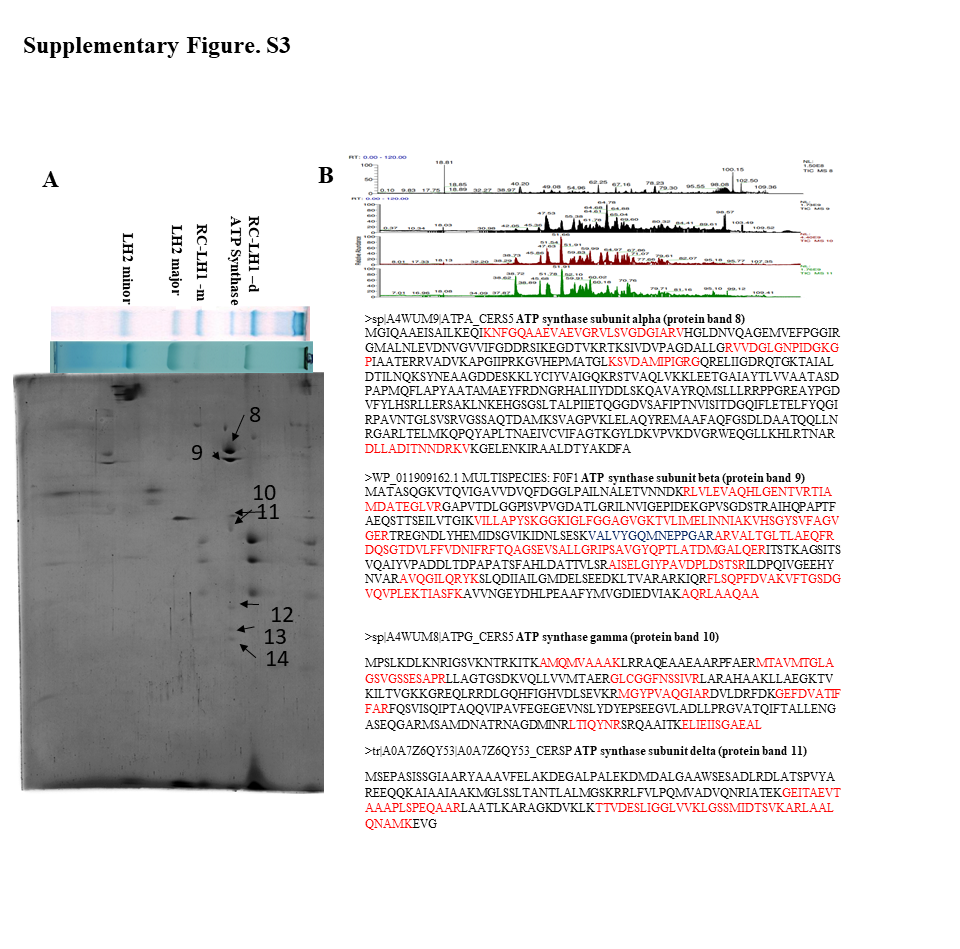

Supplement: Supplementary file 1 [file Data_Sheet_1.zip › fmicb.2024.1360650/Image 3.tif]

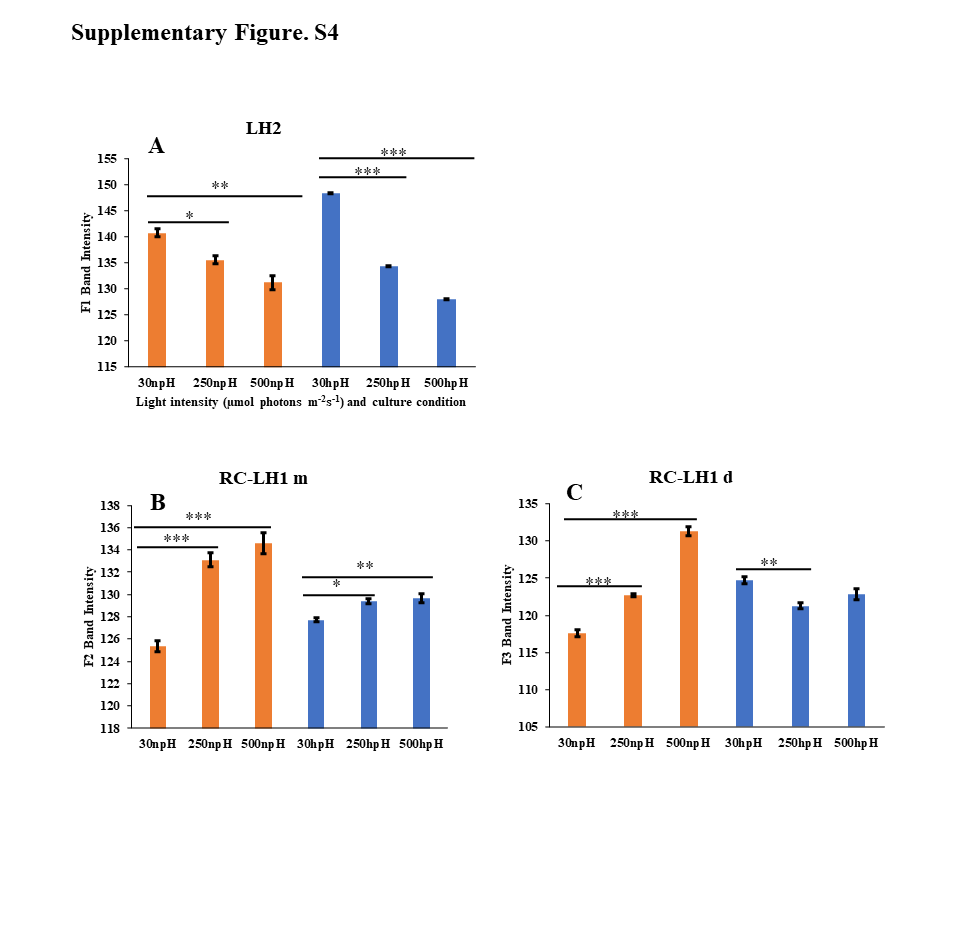

Supplement: Supplementary file 1 [file Data_Sheet_1.zip › fmicb.2024.1360650/Image 4.tif]

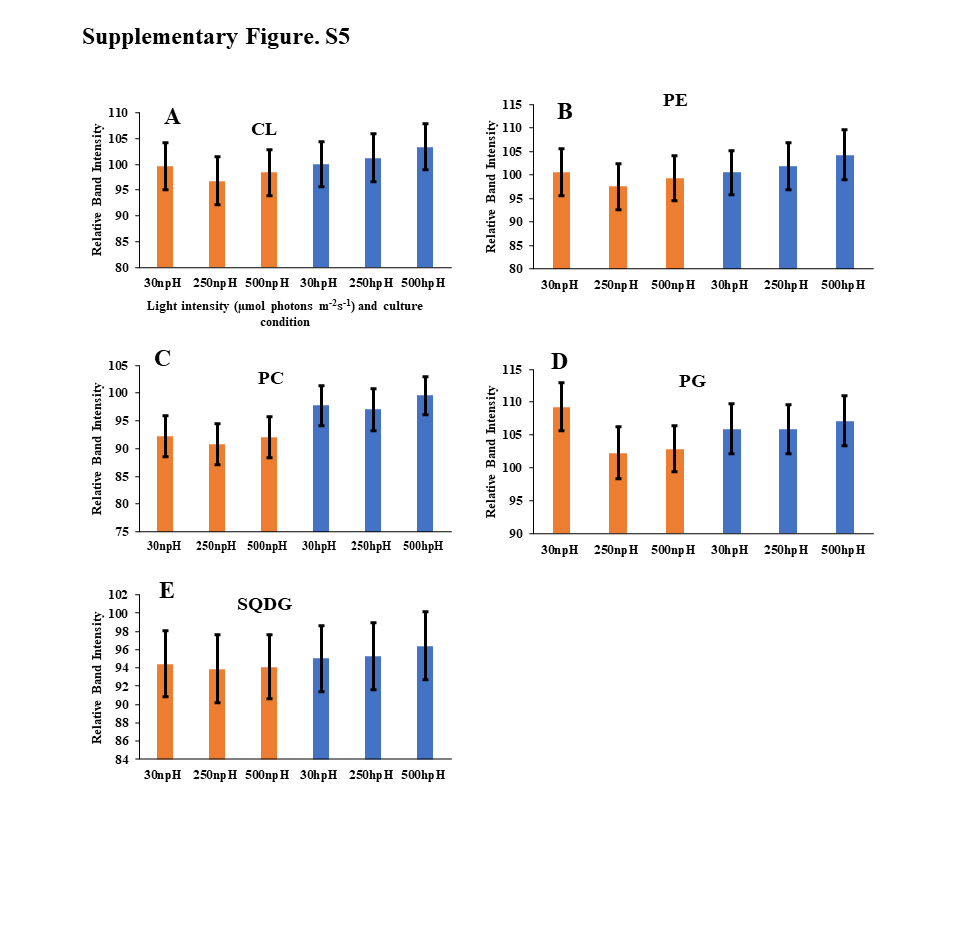

Supplement: Supplementary file 1 [file Data_Sheet_1.zip › fmicb.2024.1360650/Image 5.tif]
