# Supplementary material for: Differential stability of bacterial photosynthetic apparatus of Rhodobacter alkalitolerans strain JA916T under alkaline and light environment
Source: Front Microbiol. 2024 Mar 14;15:1360650. doi: 10.3389/fmicb.2024.1360650 (PMC10977657; doi:10.3389/fmicb.2024.1360650)

Supplementary Figure. S1

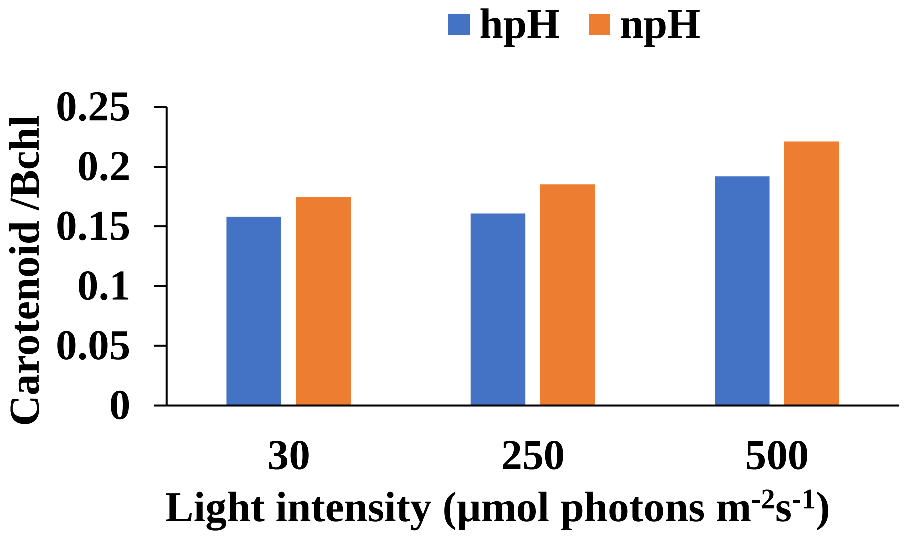

Supplementary Figure. S2

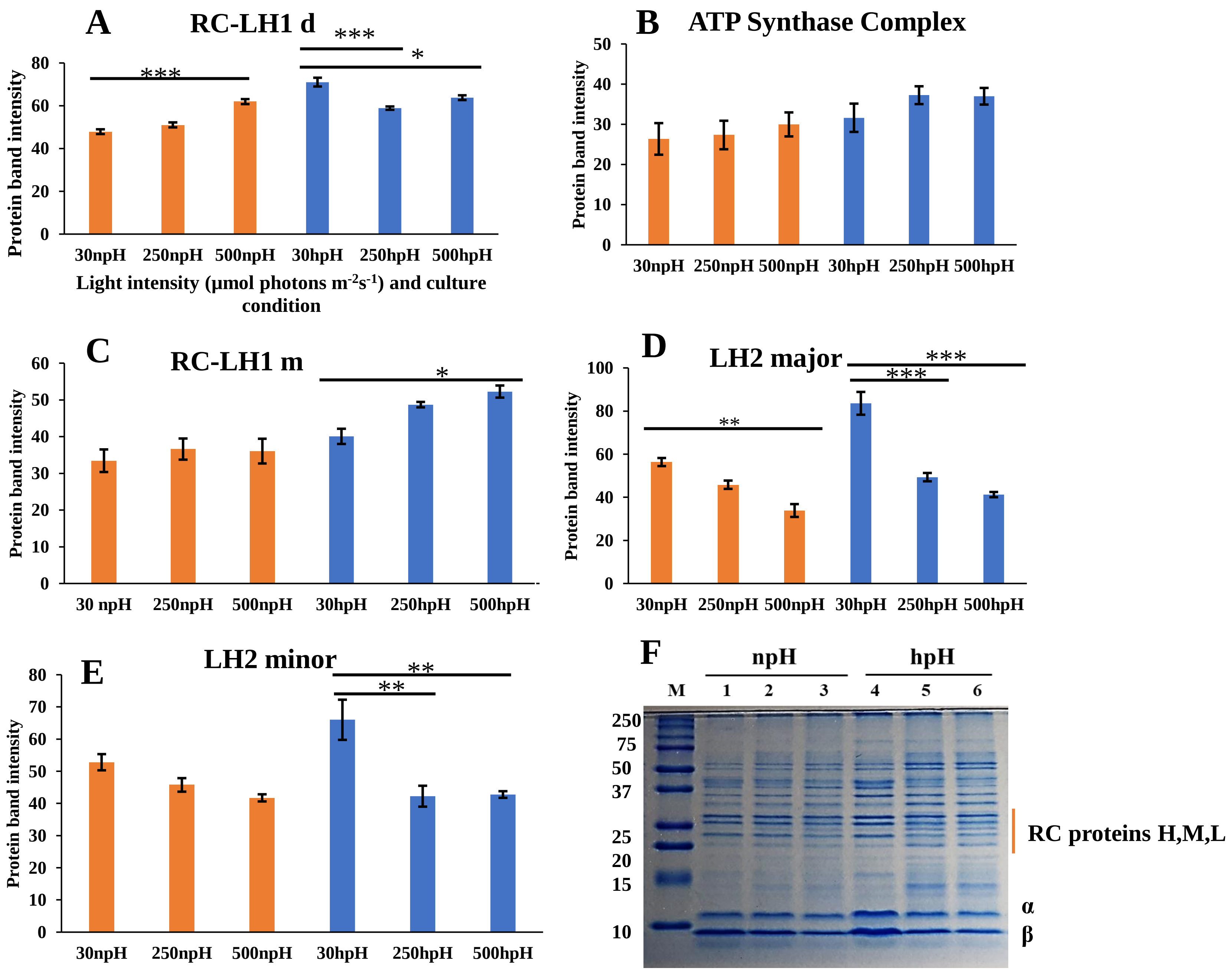

Supplementary Figure. S3

A

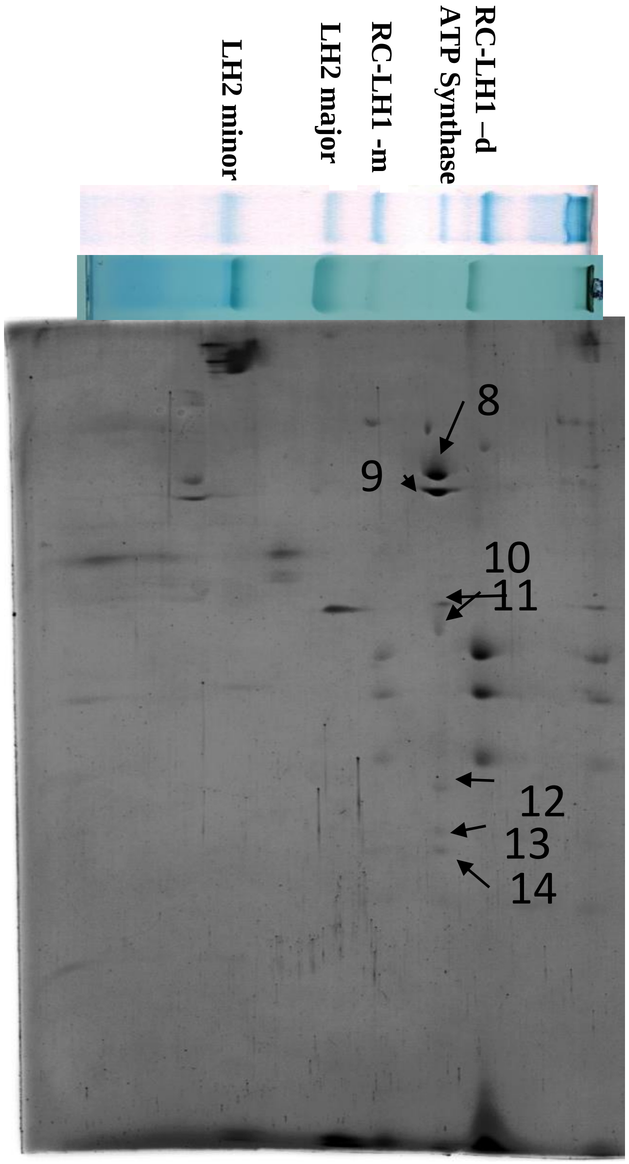

B

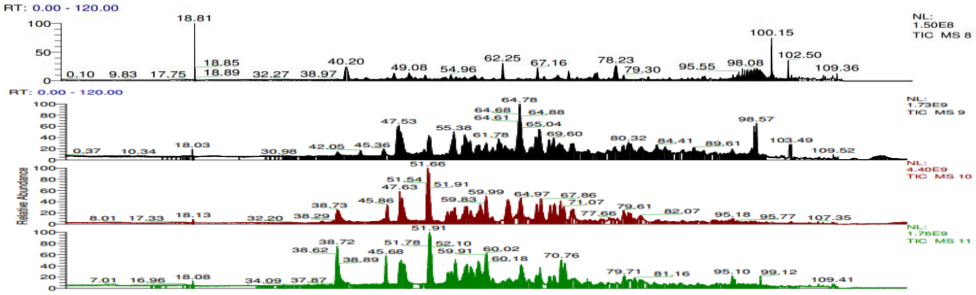

>sp|A4WUM9|ATPA_CERS5 **ATP synthase subunit alpha (protein band 8)**
MGIQAAEISAILKEQIKNFGQAAEVAEVGRVLSVGDGIARVHGLDNVQAGEMVEFPGGIR
GMALNLEVDNVGVVIFGDDRSIKEGDTVKRTKSIVDVPAGDALLGRVVDGLGNPIDGKG
PIAATERRVADVKAPGIIPRKGVHEPMATGLKSVDAMIPIGRGQRELIIGDRQTGKTAIAL
DTILNQKSYNEAAGDDESKKLYCIYVAIGQKRSTVAQLVKKLEETGAIAYTLVVAATASD
PAPMQFLAPYAATAMAEYFRDNGRHALIIYDDLSKQAVAYRQMSLLLRRPPGREAYPGD
VFYLHSRLLERSAKLNKEHGSGSLTALPIIETQGGDVSAFIPTNVISITDGQIFLETELFYQGI
RPAVNTGLSVSRVGSSAQTDAMKSVAGPVKLELAQYREMAAFAQFGSDLDAATQQLLN
RGARLTELMKQPQYAPLTNAEIVCVIFAGTKGYLDKVPVKDVGRWEQGLLKHLRTNAR
DLLADITNNDRKVKGELENKIRAALDTYAKDFA

>WP_011909162.1 MULTISPECIES: F0F1 **ATP synthase subunit beta (protein band 9)**
MATASQGKVTQVIGAVVDVQFDGGLPAILNALETVNNDKRLVLEVAQHLGENTVRTIA
MDATEGLVRGAPVTDLGGPISVPVGDATLGRILNVIGEPIDEKGPVSGDSTRAIHQPAPTF
AEQSTTSEILVTGIKVILLAPYSKGGKIGLFGGAGVGKTVLIMELINNIAKVHSGYSVFAGV
GERTREGNDLYHEMIDSGVIKIDNLSESKVALVYGQMNEPPGARARVALTGLTLAEQFR
DQSGTDVLFFVDNIFRFTQAGSEVSALLGRIPSAVGYQPTLATDMGALQERITSTKAGSITS
VQAIYVPADDLTDPAPATSFAHLDATTVLSRAISELGIYPAVDPLDSTSRILDPQIVGEEHY
NVARAVQGILQRYKSLQDIIAILGMDELSEEDKLTVARARKIQRFLSQPFDVAKVFTGSDG
VQVPLEKTIASFKAVVNGEYDHLPEAAFYMVGDIEDVIAKAQRLAAQAA

>sp|A4WUM8|ATPG_CERS5 **ATP synthase gamma (protein band 10)**

MPSLKDLKNRIGSVKNTRKITKAMQMVAAAKLRRAQEAAEAARPFAERMTAVMTGLA
GSVGSSESAPRLLAGTGSDKVQLLVVMTAERGLCGGFNSSIVRLARAHAAKLLAEGKTV
KILTVGKKGREQLRRDLGQHFIGHVDLSEVKRMGYPVAQGIARDVLDRFDKGEFDVATIF
FARFQSVISQIPTAQQVIPAVFEGEGEVNSLYDYEPSEEGVLADLLPRGVATQIFTALLENG
ASEQGARMSAMDNATRNAGDMINRLTIQYNRSRQAAITKELIEIISGAEAL

>tr|A0A7Z6QY53|A0A7Z6QY53_CERSP **ATP synthase subunit delta (protein band 11)**

MSEPASISSGIAARYAAAVFELAKDEGALPALEKDMDALGAAWSESADLRDLATSPVYA
REEQQKAIAAIAAKMGLSSLTANTLALMGSKRRLFVLPQMVADVQNRIATEKGEITAEVT
AAAPLSPEQAARLAATLKARAGKDVKLKTTVDESLIGGLVVKLGSSMIDTSVKARLAAL
QNAMKEVG

Supplementary Figure. S4

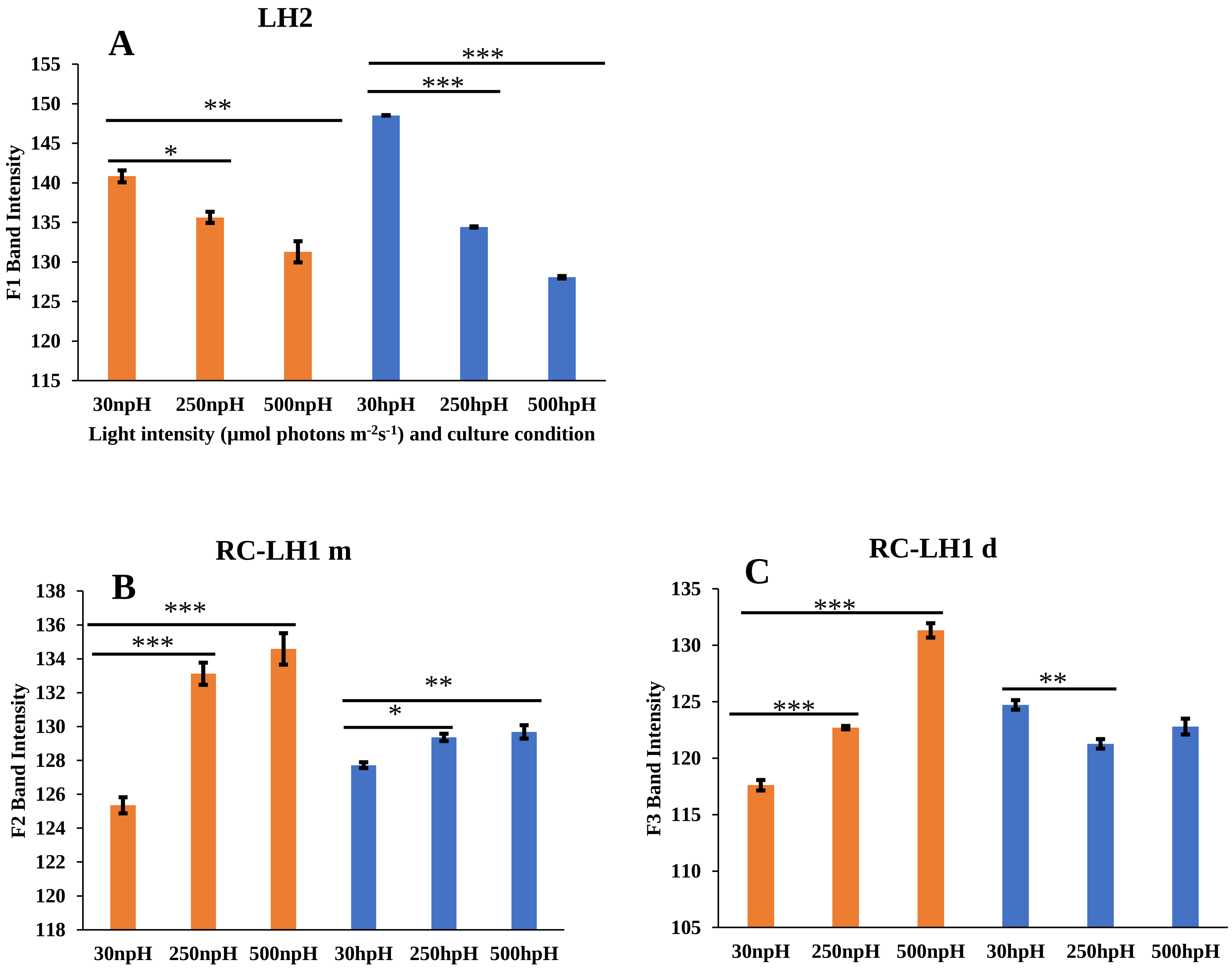

Supplementary Figure. S5

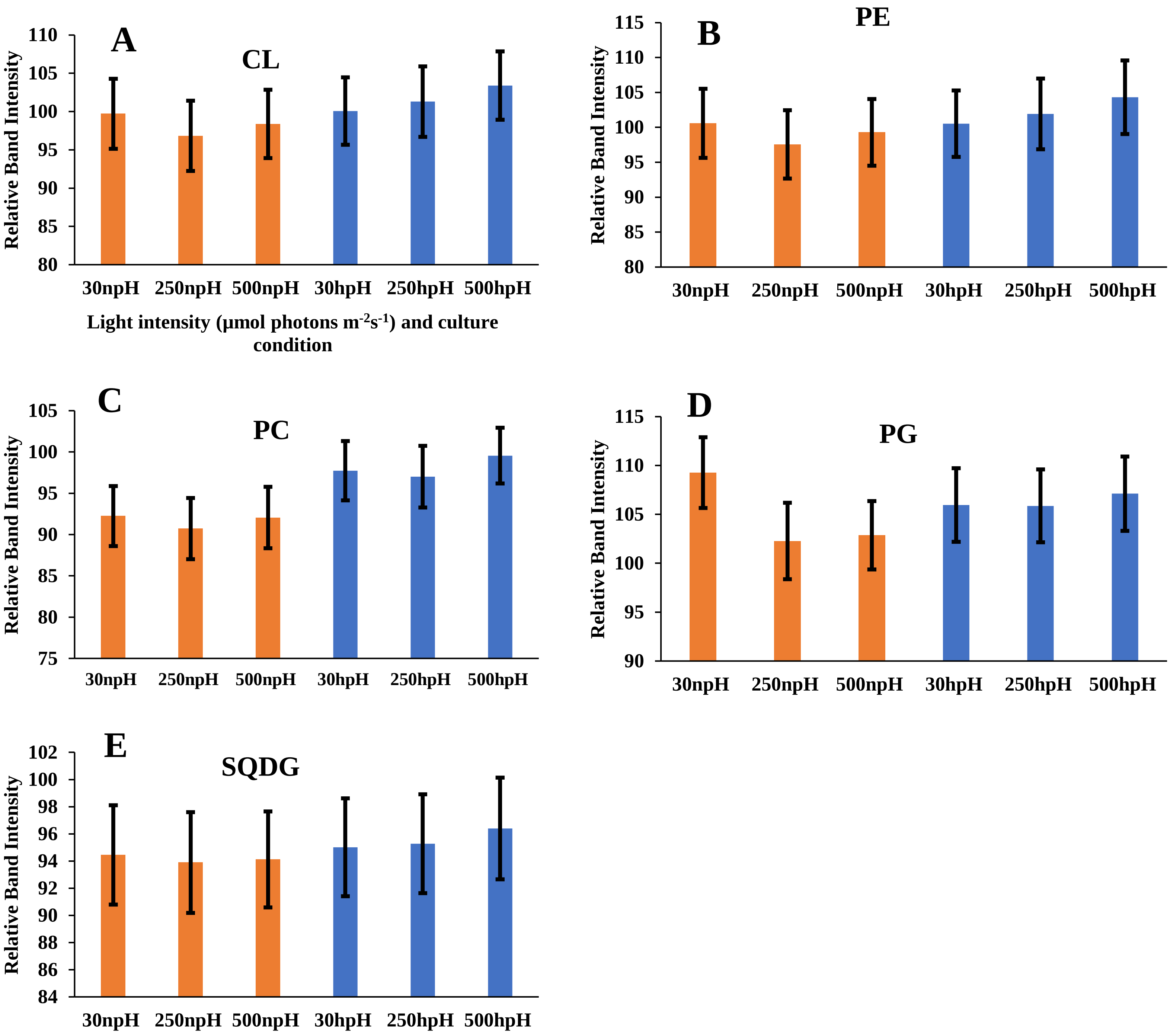

Supplement: Supplementary file 1 [file Data_Sheet_1.zip › fmicb.2024.1360650/Data Sheet 1.zip!/Supplementary Figures S1-S5.pdf]
